# Machine and deep learning approaches to understand and predict habitat suitability for seabird breeding

**DOI:** 10.1002/ece3.10549

**Published:** 2023-09-17

**Authors:** Antonio Garcia‐Quintas, Amédée Roy, Christophe Barbraud, Hervé Demarcq, Dennis Denis, Sophie Lanco Bertrand

**Affiliations:** ^1^ Institut de Recherche pour le Développement (IRD) MARBEC (Université de Montpellier, Ifremer, CNRS, IRD) Sète France; ^2^ Centro de Investigaciones de Ecosistemas Costeros (CIEC) Cayo Coco Cuba; ^3^ Centres d'Etudes Biologiques de Chizé UMR7372 Centre National de la Recherche Scientifique Villiers en Bois France; ^4^ Museo Nacional de Historia Natural La Habana Cuba

**Keywords:** animal habitat modeling, convolutional neural networks, gulls and terns breeding, remote sensing, seabirds' colonies, selection pattern

## Abstract

The way animals select their breeding habitat may have great impacts on individual fitness. This complex process depends on the integration of information on various environmental factors, over a wide range of spatiotemporal scales. For seabirds, breeding habitat selection integrates both land and sea features over several spatial scales. Seabirds explore these features prior to breeding, assessing habitats' quality. However, the information‐gathering and decision‐making process by seabirds when choosing a breeding habitat remains poorly understood. We compiled 49 historical records of larids colonies in Cuba from 1980 to 2020. Then, we predicted potentially suitable breeding sites for larids and assessed their breeding macrohabitat selection, using deep and machine learning algorithms respectively. Using a convolutional neural network and Landsat satellite images we predicted the suitability for nesting of non‐monitored sites of this archipelago. Furthermore, we assessed the relative contribution of 18 land‐ and marine‐based environmental covariates describing macrohabitats at three spatial scales (i.e. 10, 50 and 100 km) using random forests. Convolutional neural network exhibited good performance at training, validation and test (F1‐scores >85%). Sites with higher habitat suitability (*p* > .75) covered 20.3% of the predicting area. Larids breeding macrohabitats were sites relatively close to main islands, featuring sparse vegetation cover and high chlorophyll‐*a* concentration at sea in 50 and 100 km around colonies. Lower sea surface temperature at larger spatial scales was determinant to distinguish the breeding from non‐breeding sites. A more comprehensive understanding of the seabird breeding macrohabitats selection can be reached from the complementary use of convolutional neural networks and random forest models. Our analysis provides crucial knowledge in tropical regions that lack complete and regular monitoring of seabirds' breeding sites.

## Graphical abstract

Understanding seabird breeding habitat selection and suitability is often complex due to the integration of land and sea features across multiple spatial scales. A complementarity approach between convolutional neural network (CNN) and random forest (RF) models was applied to predict and explain the selection pattern of tropical larids breeding macrohabitat, highlighting the potential of this approach to be extended to other species and scenarios.

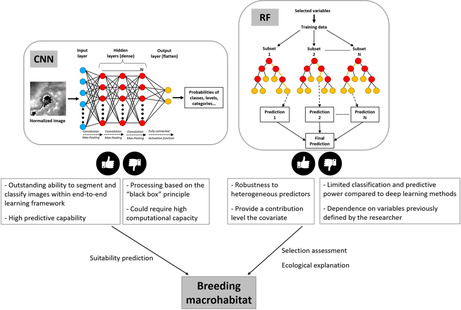

## 1 INTRODUCTION

The quality of environment in breeding habitats may greatly affect animal fitness (Danchin et al., 1998). Thus, individuals are under strong selective pressure for optimal breeding habitats (Orians & Wittenberger, 1991; Piper, 2011). This complex selection process involves environmental conditions over a large range of spatial scales and relies on hierarchical and sequential decision‐making by animals (Block & Brennan, 1993).

Several factors have been suggested for explaining how seabirds choose a place to breed: geographical features of the nesting area (area, spatial isolation) (Greer et al., 1988; Orians & Wittenberger, 1991); vegetation characteristics (coverage, height, density) (Muzaffar et al., 2015; Raynor et al., 2012); climate variability (temperature, rainfall, wind) (Córdoba‐Córdoba et al., 2010; Muzaffar et al., 2015) and socio‐ecological factors (competition, territoriality, predation pressure, fidelity to the breeding site, group cohesion, information exchange between individuals, colony recruitment, previous breeding experience) (Córdoba‐Córdoba et al., 2010; García Borboroglu & Yorio, 2007; Greer et al., 1988). Commonly, adult seabirds gather information on habitat quality (Doligez et al., 2002) over a range of spatial scales through prospective movements before breeding (Kristan, 2006; Ponchon et al., 2013). A range of oceanographic conditions surrounding the nesting sites may also be assessed by seabirds when selecting a place to nest: water masses characteristics (temperature, salinity), bathymetry and productivity‐related variables (chlorophyll‐*a* concentration, distance to food sources, prey availability and abundance). In particular, water mass properties and zooplankton abundance have been shown as important factors for this selection process in boobies and auklets (Oppel et al., 2015; Sorensen et al., 2009).

Existing studies on breeding habitat selection by seabirds are mostly focusing either on terrestrial habitats, where nests and colonies are installed, or on the surrounding marine areas, that birds use to forage during the breeding (e.g. García Borboroglu & Yorio, 2007; Raynor et al., 2012). Also, most of these studies focused on a single spatial scale of analysis and were often species or colony specific. More integrative (over land and seascapes), multi‐specific and multi‐scale approaches should improve our understanding of the breeding habitat selection process by seabirds. In addition to these existing limitations, seabird habitat selection in the tropics is much less understood than that of temperate and polar species. In tropical waters, primary productivity is generally low and seasonally stable compared to the cooler waters of polar and temperate regions (Hockey & Wilson, 2003; Jaquemet et al., 2008). One might therefore expect key factors for habitat selection to differ between tropical, temperate or polar seabirds, and hypothesize that tropical seabirds are comparatively more influenced by terrestrial than marine features.

Furthermore, many tropical regions lack a full and regular monitoring of seabirds' breeding sites due to economical and logistical constraints, as well as the scarcity of qualified human resources. For instance, Laridae (gulls, terns and skimmers; Winkler et al., 2020) in Cuba are the most abundant seabird group with 25 species recorded (Navarro, 2021), 36% of them breeding in the archipelago (Jiménez et al., 2009). However, information on their colonies is presently very limited: scarce records of sites, species, number of breeding pairs, and basic habitat features and breeding parameters (e.g. Acosta et al., 2022; Jiménez et al., 2009). In particular, the most important environmental variables affecting breeding habitat selection remain poorly known. In order to prioritize the areas to be monitored, an important prerequisite is to predict potential breeding sites as well as to identify the main drivers of breeding habitat selection at the scale of the entire archipelago. Considering both terrestrial and marine areas should provide a more realistic and eco‐functional approach to predict tropical seabirds' breeding sites.

The synergy of the aforementioned characteristics (dependence on terrestrial and marine factors, multi‐scale influence, multi‐species breeding) makes the study of seabird breeding macrohabitat (i.e. the breeding location such as island, peninsula, beach) complex. Apparently, few tools have enough potential to assess, holistically, the suitability and selection of these macrohabitats. But a solution could be found within the field of machine learning, a family of artificial intelligence tools that aims to learn functional relationships from data (Borowiec et al., 2022; Fincham et al., 2020; Olier et al., 2021). Since their dissemination in the 1990s, machine learning models have shown marked statistical and predictive superiority over classical approaches, such as the maximum likelihood estimation and null hypothesis significance testing (Pichler & Hartig, 2023).

Among the most popular models are the neural networks (such as convolutional neural networks [CNN]), and random forest (RF). CNNs belong to the deep learning subfield and have become a state‐of‐the‐art approach in the field of computer vision and remote sensing (Borowiec et al., 2022; Ghanbari et al., 2021; Ma et al., 2019). CNNs are composed of multiple layers of processing units which can learn from complex features and represent data with a high level of abstraction at multiple scales. They are known for their outstanding ability to segment and classify images within end‐to‐end learning framework, i.e. without requiring any preliminary feature engineering (Fincham et al., 2020; Kattenborn et al., 2021; Ma et al., 2019). RF (Breiman, 2001) is highlighted for its robustness to heterogeneous predictors, its high accuracy (Ma et al., 2019) and its ability to provide a contribution level or importance of each covariate. CNNs usually outperform RFs for classification and prediction purposes (Kattenborn et al., 2021; Mahdianpari et al., 2018). Yet, an important advantage of RFs over CNNs is their more explicit understanding of the associations between the response variable and its covariates. In the remote sensing research area, CNNs have been identified as potentially well‐suited to prediction of habitat suitability for animals such as birds (e.g. Chilson et al., 2019; Su et al., 2018). “A picture is worth a thousand words” and a satellite image represents an excellent example of that due to its stack of spectral bands with high potential for seabird macrohabitat description. Su et al. (2018) used CNNs (and Support Vector Machine) with satellite images to model the habitat suitability for a migratory geese species. Others, as Chilson et al. (2019) and Wang et al. (2021), identified birds' habitat elements using radar data and photographic images, respectively. Deneu et al. (2021) used CNNs to improve species distribution modeling by capturing complex spatial structures of the environment.

**FIGURE 1:**
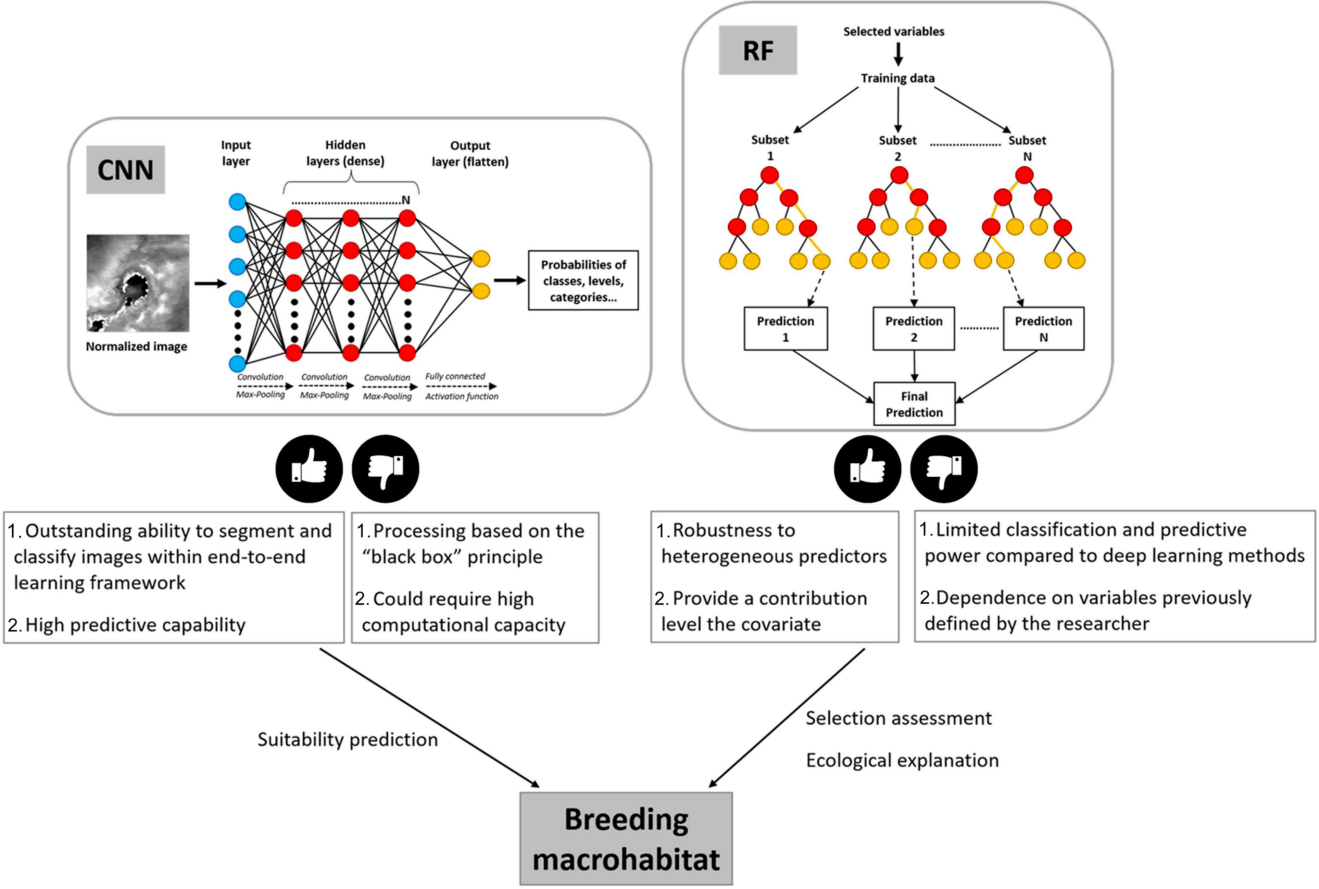
Complementarity approach between convolutional neural networks and random forests models to study seabird breeding macrohabitat.

Despite the development of some alternative procedures, the main limitation of CNNs (and deep learning in general) is that they operate as a “black box,” which prevents the ecological interpretation of the processes under study (Borowiec et al., 2022; Pichler & Hartig, 2023). However, considering the main strengths of CNN (high performance for prediction) and RF (assessing of ecological hypotheses through the covariates contribution), the complementary use of both methods could increase our understanding of the patterns and processes involved in macrohabitat selection and be helpful for developing effective management and conservation strategies (Figure 1). Here, we predict the suitability of macrohabitat for the breeding of Laridae in Cuba (using CNN) and investigate the ecological variables driving their habitat selection (using RF) from satellite data. More precisely we (1) predict the breeding macrohabitat suitability of Laridae at the scale of the entire Cuba archipelago using CNN, and (2) assess the selection of the breeding macrohabitat by these seabirds considering the contribution of landscape and seascape covariates, at different spatial scales, using RFs.

## 2 MATERIALS AND METHODS

### 2.1 Study area

**FIGURE 2:**
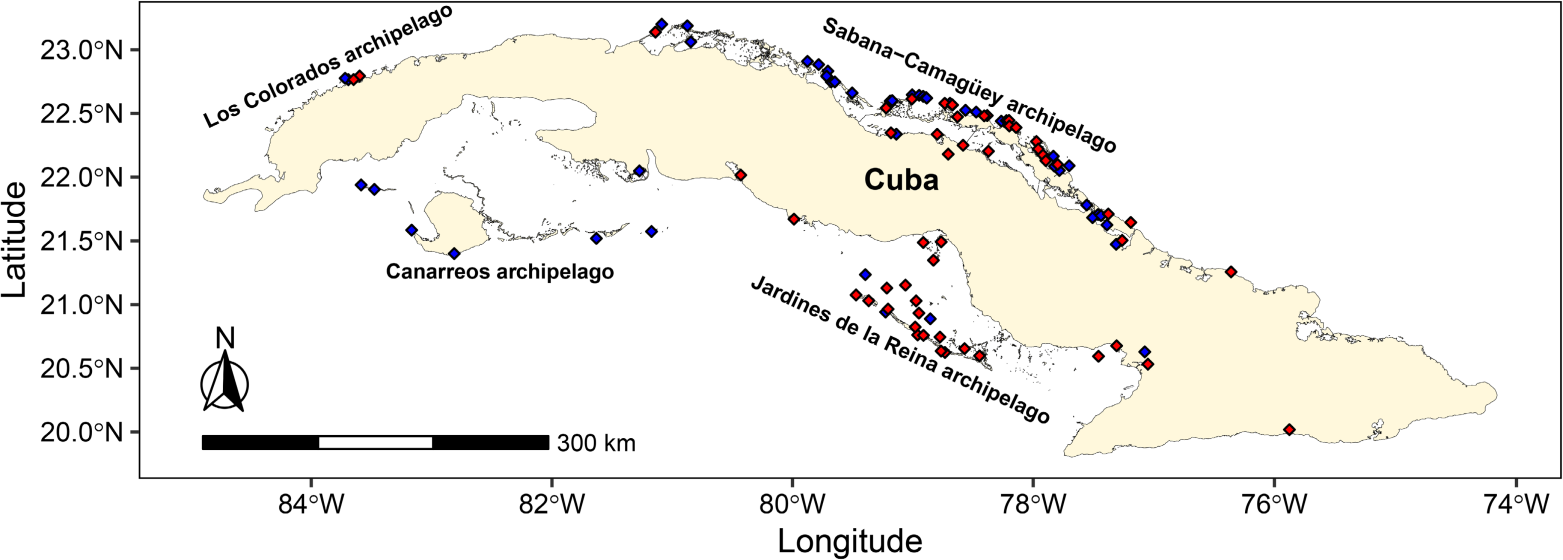
Map of the study area and the sites used to train and validate the modeling of the breeding macrohabitat suitability of nine Laridae species. Blue diamonds = breeding sites, red diamonds = non‐breeding sites.

This study focuses on the marine coastal ecosystems of the Cuban archipelago (Figure 2). Cuba is the largest Caribbean island (length = 1256.2 km, maximal width = 191 km) and includes four insular groups (Los Colorados, Sabana‐Camagüey, Canarreos and Jardines de la Reina) featuring >1600 cays (small, low‐elevation, sandy islands on the surface of the coral reef) and islets with large variation in relief, geology and landscapes. Climate is tropical hot and seasonally wet with marine influence and semi‐continental traits (www.insmet.cu). Annual mean temperature varies between 24°C in the plains of the main island and >34°C at the eastern coasts. Mean relative humidity in the island is high (≈82%–90%) and mean annual precipitation ≈1375 mm. Daily weather variations are more important between November and April while the weather is more stable during May–October due to the influence of a North Atlantic anticyclone (www.insmet.cu). The mean sea surface temperature over the continental shelf varies from ~23 to 28°C in January and from ~29 to 32°C in September, from North to South, with the largest spatial gradients at the vicinity of the shelf break. The mean chlorophyll‐*a* varies from ~0.5 to >10 mg m^−3^ with the largest values observed between the coast and the northern islands as well as in the Southwest region, with moderate seasonal variations.

#### 2.1.1 Breeding and available sites

We compiled and filtered (deletion of duplicates, erroneous and imprecise data about species identification, location of colonies and date of breeding) all available informations on observed breeding sites of Laridae (i.e. cay, islet or coastal site) from scientific publications, books, thesis, project reports and unpublished data. A database was built with the names and spatial coordinates of the 49 reported breeding sites (Figure 2), years of observation (1980–2020), breeding species and information sources (Table S1). Observed breeding species of Laridae were Laughing Gull *Leucophaeus atricilla* (LAGU), Brown Noddy *Anous stolidus* (BRNO), Sooty Tern *Onychoprion fuscatus* (SOTE), Bridled Tern *Onychoprion anaethetus* (BRTE), Least Tern *Sternula antillarum* (LETE), Gull‐billed Tern *Gelochelidon nilotica* (GBTE), Roseate Tern *Sterna dougallii* (ROST), Royal Tern *Thalasseus maximus* (ROYT) and Sandwich Tern *Thalasseus sandvicensis* (SATE). Breeding records of Common Tern *Sterna hirundo* were treated as ROST due to the misidentification of these species' colonies (Navarro, 2021; Nisbet, 2020). Additionally, we selected (non‐randomly) 52 sites distributed along the coast of Cuba where none of the nine species was observed breeding in 2020 (Figure 2, Table S1). These non‐breeding sites represented the potential macrohabitat available. Both terrestrial and marine features surrounding the observed breeding sites were considered for predicting suitable breeding macrohabitats.

### 2.2 Acquisition and formatting of breeding macrohabitat data

#### 2.2.1 Satellite images

For each breeding and available site we extracted satellite imagery of Landsat 5 and 7 (Table S2) from EOS Data Analytics platform (https://eos.com). The date of the image was matched to the year of the breeding colony presence record, while images from 2020 were used for non‐breeding sites. Several images were associated to each site (depending on availability) to ensure a good representation of the natural variability during the breeding period (May–August) and to reduce the influence of clouds in some images. In some cases, we incorporated images of both months of April and September (climatology similar to the May–August period) when none was available from May to August of the current year. In total, we selected 136 satellite images describing the conditions of the study sites (Table S2).

We then resized the Landsat images into scenes (9.0 × 9.0 km square areas centered on the sites) and standardized them through the “Dark Subtraction” (based on the bands minimum digital number) to apply atmospheric scattering corrections to the imagery data and “SLC Gap‐Filled” correction for Landsat 7 imagery since 31 May 2003. In the end, GeoTIFF files (299 × 299 pixels, 30 m‐spatial resolution) were created that included the visible and infrared bands. Panchromatic and thermic bands were excluded because these do not match between Landsat satellites. The definition of scene size followed a balanced criterion: sufficiently large image size that included land and sea components, and sufficiently small to minimize the inclusion of other breeding and available sites in the same image scene (which would affect the prediction quality). We organized the data into two datasets considering the quality of scene images related with cloud cover (Table S3) to control for cloud‐related confounding effects. We randomly mixed the images database of both types of sites (i.e. with and without breeding colonies) and then split them into two groups containing 70% (training) and 30% (validation) of the data (Table S3) for the building and selection of the best CNN.

Satellite images from 2021 at 12 breeding and 52 non‐breeding sites (verified as such that year) were used as test dataset to assess the predictive performance of the CNN. For predicting breeding macrohabitat suitability, we applied the same preprocessing to Landsat 7 images of 2021 (Table S2) that covered the entire Cuban archipelago. A mosaic was built with these images, masking mainland to retain only the marine‐coastal ecosystems up to the insular shelf. Images with open water only or predominance of land of the main two islands (Cuba and Juventud) were excluded since they were irrelevant to Laridae breeding (Jiménez et al., 2009). Finally, this mosaic image was gridded into 793 scenes (63,805.5 km^2^) with the same format and structure than train and validation datasets. Satellite images were processed using the ENVI 4.7 (ITT VIS Inc) software.

#### 2.2.2 Physical and geographical covariates

Because of the absence of information on the foraging ranges of Cuban seabirds during their breeding period, we did a bibliographic compilation of all information available on the same species observed elsewhere during breeding, from polar to tropical zones. From this review, we estimated the potential maximum foraging ranges during breeding for each studied species (Table S4). Then, we defined three spatial scales (radius of 10, 50 and 100 km from the breeding site) approximately corresponding to the estimated foraging ranges of our study species, and computed several oceanographic characteristics at each spatial scale.

Twelve potentially important features for the establishment of Laridae breeding colonies, described through 18 metrics, were considered at the defined spatial scales (Table 1): 11 of them described the conditions of the nesting landscape, and seven of them described the conditions of the surrounding seascape. The variables were measured at the date of the colony observation, and in 2020 for the non‐breeding sites.

**TABLE 1:** Potentially important variables for Laridae breeding macrohabitat selection (breeding site, i.e. cay, islet or coastal site) in Cuba.

Feature	Variable	Unit	Ecological meaning
Site extent	Area	km^2^	Available space for nests establishment
Site perimeter	Perimeter	km	Indicator of the availability of potential coastal zones for breeding
Site shape	Shape index based on perimeter/area ratio	–	Related to geographical features (e.g. peninsulas) that could be important for breeding
Isolation degree	Minimum distance from the colony to the nearest cay/islet	km	Indicator of accessibility for predators and other disturbance sources
	Minimum distance from the colony to the nearest main island (Cuba or Isla de la Juventud) of the archipelago	km	Indicator of accessibility for predators and high disturbance sources (higher risk)
	Cays/islets number within 10, 50 and 100 km of the colony	–	Indicator of the number of potential sources (at different spatial scales) of predators, alien species and other disturbances that could affect the colonies
Terrain	Non‐flooding zone cover	%	Suitability of the locality for colony establishment based on flooding risk
Vegetation	Vegetal cover_total_	%	Surface occupied by plants (species‐specific suitability for breeding, Burger & Gochfeld, 1981; Raynor et al., 2012)
	Vegetal cover_moderate + dense_	%	Surface occupied by moderate to dense vegetation that could affect the establishment of colonies (some Laridae tend to avoid high vegetation cover while others are attracted, Bukacinska & Bukacinsky, 1993; Burger & Gochfeld, 1986)
Oceanographic	Sea surface temperature within 10, 50 and 100 km radius of the colony	°C	Reflects thermal conditions that influence primary productivity and prey availability at different foraging ranges
Bathymetric	Minimum distance to the 200‐m isobath	km	Indicates the limit of the insular shelf in Cuba and therefore is a proxy for suitable foraging areas for most Laridae (Schreiber & Burger, 2002).
Phytoplanktonic biomass	Chlorophyll *a* concentration at sea surface within 10, 50 and 100 km radius of the colony	mg m^−3^	Proxy for phytoplanktonic biomass, primary productivity and prey availability at different foraging ranges

Using the Landviewer product of the EOS Data Analytics platform, we calculated the Normalized Difference Water (Gao, 1996) and Vegetation (Rouse et al., 1973) Indexes (NDWI and NDVI respectively) from satellite images (using Green, Red and Near Infrared bands) of the Landsat series. Dates of images ranged between May and August, matching the breeding season of Laridae in Cuba (Jiménez et al., 2009). Both spectral indices vary between −1 and 1 with higher numbers corresponding to higher humidity (NDWI) or green vegetation (NDVI). Based on the NDWI we then calculated the drought emerged areas (NDWI range = −1 to 0.2) and the percentage of non‐flooding cover (NDWI range = −1 to 0). The NDVI allowed to quantify the total (NDVI range = 0.2–1) moderate and dense vegetation covers (NDVI range = 0.4–1). Thus, the area, non‐flooding zone cover and vegetal covers of each nesting site were computed from the satellites images. The perimeter was calculated after vectorization of the imagery scenes.

We calculated the index of shape complexity for islets and cays (Hu et al., 2011) as SI = *P*/[2 × (*π* × *A*)^1/2^], where SI = shape index, *P* = perimeter and *A* = area of the site. A SI value of 1 indicates an islet or cay with a perfect circular shape and SI increases as the shape becomes more irregular and complex. Isolation variables (minimum distances to Cuba/Isla de la Juventud (IJ) and to nearest cay, and cays/islets number at the three spatial scales) were estimated using Google Earth Pro 7.3.3 software. Minimum distances to the 200‐m isobath were measured using a bathymetric shapefile of the exclusive economic zone of Cuba (using information both from GEBCO and Cuban research agencies databases).

Sea surface temperature (SST) and surface chlorophyll *a* concentration (Chl *a*) around the sites were obtained at a spatial resolution of 1 km from level‐2 MODIS‐Aqua satellite data (https://oceancolor.gsfc.nasa.gov/data/aqua/) after sampling and spatial reprojection of the data. For both variables we averaged the monthly values between May and August of the year corresponding to the last register of each breeding colony and for 2020 for available sites. Nevertheless, due to the absence of logistical support for a systematic monitoring of these variables before 2002, and given the small interannual variability of both variables in Cuba, data for breeding colonies observed in that period were estimated from the mean of 2002–2021 period for the same months (Figure S1). A summary of the satellite images used for the study is provided in Table S2.

The eldest colony record, at Rincón del Guanal (Table S1), was excluded from the study as we could not obtain the same variables from Landsat 4 satellite (fewer bands than Landsat 5 and 7). Breeding macrohabitat was characterized considering both mixed and monospecific colonies.

### 2.3 Prediction and selection assessment of the breeding macrohabitats

#### 2.3.1 CNN implementation

A CNN architecture is typically composed by multiple layers of processing units where two main processes occur: convolution and pooling. During convolutions several filters are applied to extract relevant features of data that will be used for calculating the matches in the testing phase. Pooling operations capture large images and reduces the parameters to preserve important information. Kattenborn et al. (2021) and Krishna and Kalluri (2019) offer more details about architectures, parameters and functions of CNNs. Here, we use a CNN with three consecutive layers of convolutions and max pooling, followed by a dense network. Finally, the last layer consists in a sigmoid activation so that the output of the network is a value between 0 and 1. This CNN aims therefore to ingest the selected bands of Landsat satellite images of 299 × 299 pixels (Table S3) as input data and to output the probability of the described habitat to be suitable for breeding seabirds.

Parameters were finally estimated using an Adam optimizer and minimizing a “Sparse categorical crossentropy” loss. In order to prevent overfitting, we also added a L2 regularizer, a Deep Learning technique to get better generalization and predictive properties (Kussul et al., 2017). Finally, models were trained using the “training dataset,” and selected when minimizing accuracy score over the “validation dataset.” This analysis was implemented with the keras R package (v. 2.6.1) (Kalinowski et al., 2021).

We performed this training procedure for distinct parameters and the best CNN (better performance and lowest loss in validation and test datasets) was used to predict the breeding macrohabitat suitability using the satellite images of 2021 along all marine‐coastal ecosystems of the archipelago. The most frequently reported metrics, Overall Accuracy, Precision, Recall and F1‐score (Table S5) were used to assess CNN performance. All analysis was implemented in R 4.1.1 (R Core Team, 2021).

#### 2.3.2 RF implementation and contribution of variables

Variables were compared among sites using Mann–Whitney *U*‐tests considering significance at *p* < .01. Breeding macrohabitat selection was analyzed through random classification forests (RFs) models considering the measured variables at three spatial scales. Similar to CNN, we mixed and split the data to create the training and validation datasets (70% vs. 30% proportions) for the building and selection of the best RF. The same training and validation datasets were used in all RFs to compare their classification performances. We implemented three RFs, one that processed physical‐geographical variables registered within a radius of 100 km from the breeding colony (RF_100; 300 trees and four variables by split), a second within a 50 km radius (RF_50; 200 trees and four variables by split) and a third one within a 10 km radius (RF_10; 150 trees and three variables by split). For each RF type we used the 10 best runs in order to get an average and variance of model performance.

Same performance metrics than CNN (Table S5) were used to assess the training, validation and test of RFs. Variables contributions were calculated from the mean decreasing Gini index (values are directly proportional to variable importance) derived from the RF with better performance. The randomForest R package (v. 4.6‐14) (Breiman et al., 2018) was used to this analysis.

## 3 RESULTS

### 3.1 Prediction of macrohabitats breeding suitability

The performance of CNN was high, exhibiting indicators values ≥80.0% for the validation datasets (Table 2). Image quality (according to cloud cover) had no significant consequence on the classification power of CNNs (F1‐score validation = 85.7% and 84.6% for CNNs that used all and best images, respectively; Table S3). We thus worked with the architecture that used all images. Based on the test dataset, the CNN exhibited good performance test indicators with accuracy = 79.7%, precision = 91.5%, recall = 82.7% and F1‐score = 86.9%.

**TABLE 2:** Training and validation performance (in %) of a convolutional neural network (CNN) and three random forest (RF) models used to respectively predict breeding site suitability and assess breeding site (macrohabitat) selection by Laridae in Cuba.

Model type	Training accuracy	Training F1‐score	By image				By site			
			Validation accuracy	Validation precision	Validation recall	Validation F1‐score	Validation accuracy	Validation precision	Validation recall	Validation F1‐score
CNN	98.4	98.7	75.7	75.5	76.9	76.2	86.7	80.0	92.3	85.7
RF_10	72.0 ± 2.5	71.2 ± 2.3	–	–	–	–	70.0 ± 1.6	67.4 ± 2.0	69.3 ± 3.4	68.3 ± 1.9
RF_50	81.7 ± 2.4	80.7 ± 2.4	–	–	–	–	77.3 ± 2.1	77.8 ± 3.4	72.1 ± 2.3	74.8 ± 2.0
RF_100	85.1 ± 1.1	84.5 ± 1.4	–	–	–	–	76.7 ± 0^a^	76.9 ± 0^a^	71.4 ± 0^a^	74.1 ± 0^a^

*Note*: RF_10, RF_50 and RF_100 indicate models that used physical‐geographical variables within 10, 50 and 100 km radius from localities, respectively; CNN = model that used Landsat images with 9 × 9 km square areas. Statistics indicate the mean (± standard deviation) of the best 10 runs for each RF.

^a^ There was no variability in the indicator.

**FIGURE 3:**
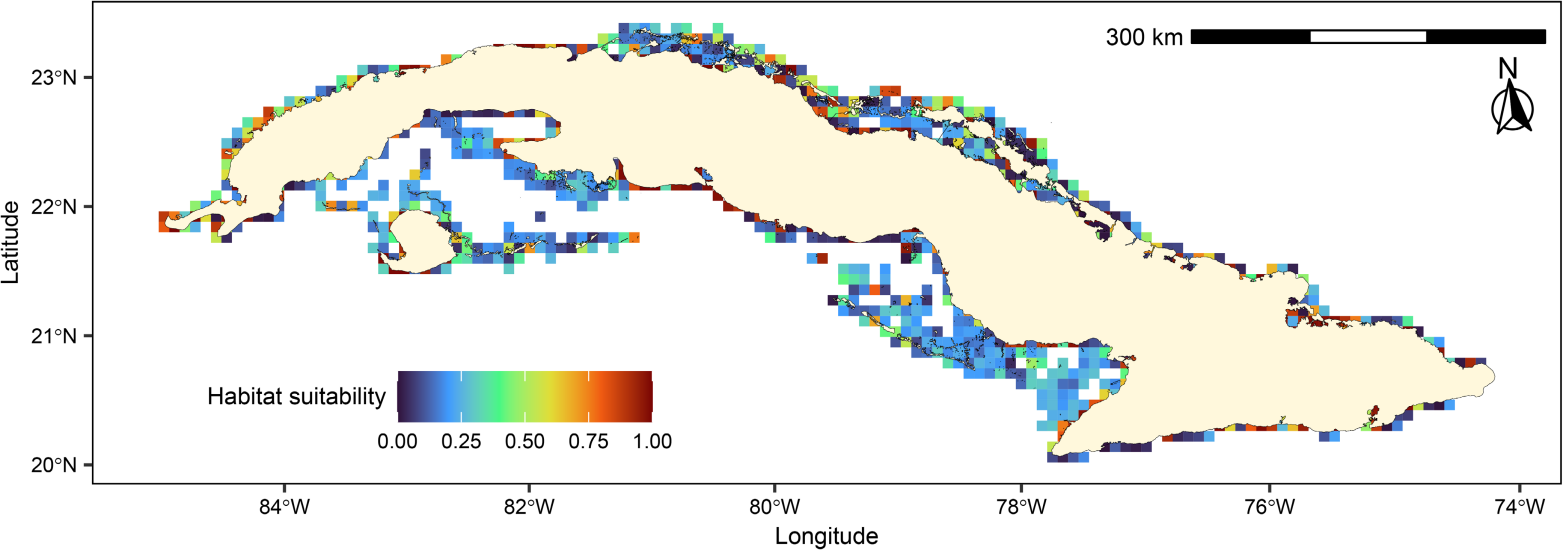
Convolutional neural network prediction of macrohabitat suitability for the breeding of Laridae in Cuba, for the 2021 breeding season, using Landsat images.

When used for predicting over the entire Cuba archipelago, the CNN estimated 32,184, 12,069, 6598 and 12,954 km^2^ of suitable habitat for breeding within 0–.25, .26–.50, .51–.75 and .76–1 probability ranges, respectively (Figure 3). Thus, the probability ranges >.50 and >.75 covered 30.6% and 20.3% of the predicted area, respectively. The best areas (high suitability scores) tended to be concentrated in three general types of ecosystems: (1) remote cays/reef islets of all subarchipelagos (Figure 2, with Jardines de la Reina archipelago under‐represented), (2) coastal zones with sand, rocks or interior lagoons and (3) interior of bays, gulfs and swamps that contained small islets and sand banks (Figure 3). The southern region of Cuba had less suitable breeding macrohabitats than the northern region (Figure 3).

### 3.2 Breeding macrohabitat selection and importance of covariates

**FIGURE 4:**
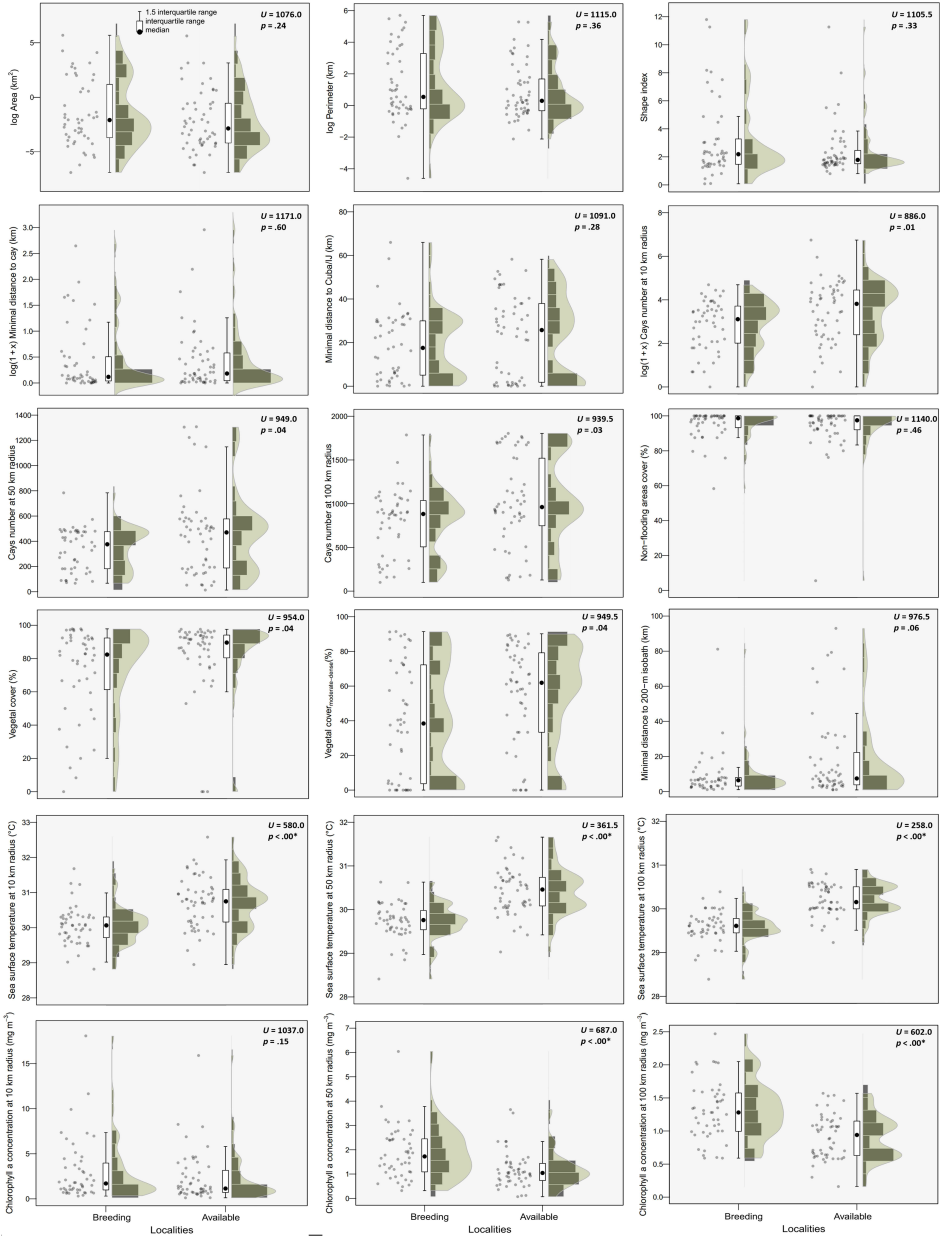
Comparison of 18 physical‐geographical variables corresponding to 48 breeding and 52 non‐breeding sites (available macrohabitats) for Laridae in Cuba. Some variables were log‐transformed for visualization purpose exclusively. *Significant differences.

**FIGURE 5:**
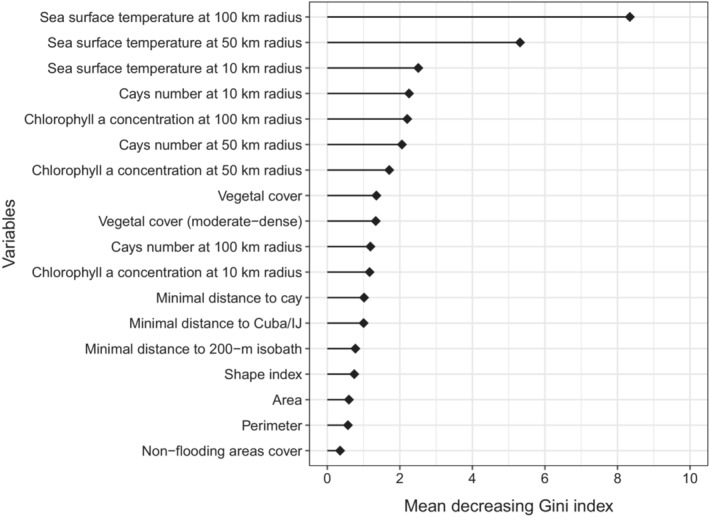
Contribution of marine and terrestrial environmental features to the breeding macrohabitat selection pattern of Laridae in Cuba based on random forests classification models (corresponding to the spatial scale of 100 km radius from breeding colonies, but includes the nested scales of 10 and 50 km radius).

The general statistics (median, quartiles) of most covariates were quite similar between breeding and non‐breeding sites (Figure 4, Table S6). Only SST at the three spatial scales and Chl *a* at 50 and 100 km radii exhibited significant differences: SST was lower and Chl *a* higher at breeding sites (Figure 4). Additionally, breeding sites tended to have a smaller number of cays/islets within 10 km compared to non‐breeding sites (*p* = .01, Figure 4). Performance metrics for RF_50 and RF_100 were very similar and outperformed RF_10 (Table 2). For RF_100, SST had the highest contribution to discriminate breeding from non‐breeding sites (Figure 5). A second group of covariates with lower contribution included isolation‐related variables (number cays/islets within 10 and 50 km from colonies) and Chl *a* within 100 and 50 km radius (Figure 5). Remaining variables exhibited relatively low contributions and non‐flooding area cover had the lowest importance (Figure 5).

## 4 DISCUSSION

### 4.1 Prediction of breeding macrohabitat suitability

According to our results, prediction of habitat suitability can be successfully obtained by processing satellite images with CNN exclusively. This constitutes a significant advance for habitat ecology studies and expands the applications and perspectives of image analysis via deep learning approaches. Deep architecture of CNNs conveys a high computation cost (Kattenborn et al., 2021) but, at the same time its versatility provides a great generalization capacity with a broad applicability in the remote sensing field (Kattenborn et al., 2021; Ma et al., 2019; Mahdianpari et al., 2018). Our CNN had relatively good performance and provided a map of macrohabitats suitability for Laridae over the whole Cuban archipelago for the year 2021. Predictions were based on physical‐geographical suitability of marine‐coastal ecosystems (from the images) but it does not imply per se the existence of breeding colonies at areas with higher habitat suitability. Deep learning algorithms, such as CNNs, do not necessarily “learn” the right causal dependencies between input features and the response (Pichler & Hartig, 2023). However, these areas represent sites with favorable conditions for breeding in 2021 and thus constitute alternatives or additional breeding sites for Laridae around Cuba.

The most suitable breeding sites (probability range >.50) for Laridae exhibited a scattered general distribution along the coasts of Cuba. Although a slightly higher concentration of sites occurred in the northern marine‐coastal ecosystems. Nevertheless, areas of Los Colorados, Canarreos and Sabana‐Camagüey archipelagos showed higher number of favorable breeding sites acting as potential hotspots for Laridae reproduction (Figures 2 and 3). These predictions are relatively consistent with current field knowledge in these areas, and with the location of historical persistent breeding colonies.

In particular, breeding habitats for Laridae such as beaches, rocky platforms and sand banks in distant or difficult‐access cays and islets were often true positive predictions. Two of the most scientifically studied regions, the Sabana‐Camagüey and Jardines de la Reina archipelagos, had respectively high and low predicted breeding habitat suitability depicted by CNN, a point confirmed by field observations (Figures 2 and 3). Nevertheless, some predicted areas seemed irrelevant for breeding because of the presence of extensive anthropogenic infrastructures (cities, towns, industries, agricultural fields) that might cause disturbance. Although this study focused on the physical‐geographical suitability of Laridae breeding macrohabitats, the satellite images processed through our CNN also contained spectral information on some anthropogenic features. This provides a more comprehensive aspect to our predictions.

On the other hand, main false negatives of the predictions were located at Mono Grande, Cinco Leguas, Felipe de Barlovento and Las Salinas breeding sites (Table S1). Prediction scenes (grid cells) containing these breeding sites were not highly different (from a visual interpretation) from the training sites. Then, this suggests the importance of incorporating oceanographic (e.g. SST, Chl *a*) or ecological variables (e.g. prey availability‐related) into the CNN to improve its prediction quality. Scene classification with an emphasis on land cover, vegetation and crop types is one of the most common applications of CNNs (e.g. Kattenborn et al., 2021; Kussul et al., 2017; Mahdianpari et al., 2018). However, these classifications are based on relatively easily distinguishable element classes (e.g. water, bare soil, marsh, fen, forest, grassland, paddy rice) whereas animal habitat suitability is a more complex phenomenon.

It is important to point out that the habitat suitability diversity predicted by the model (that addressed all Cuban Laridae) could result from the different species‐specific breeding habitats requirements; especially LETE that has a high dynamic and opportunistic behavior to select its breeding sites (this species may change it nesting sites between consecutive breeding seasons depending on the availability of isolated sand bodies). For this reason, even if CNN obtained relevant predictive performance metrics, the prediction map (Figure 3) should be interpreted carefully, and further studies would be required to improve its accuracy. However, the good global quality of the model highlights its potential for application to other species and regions of the world. Normally, CNNs are excellent tools for capturing complex hierarchical patterns from images (Borowiec et al., 2022).

### 4.2 Breeding macrohabitat selection pattern

Simplistic approaches to the study of breeding habitat selection have been criticized decades ago (e.g. Burger & Shisler, 1978) as the environment of many animals, such as seabirds, consists in heterogeneous composition of habitat characteristics along several spatial and temporal scales (Danchin et al., 1998). The abundance of food resources, microclimate and physical‐geographical attributes of the local landscape are elements usually relied upon by colonial seabirds for the selection of good breeding habitats (García Borboroglu & Yorio, 2007). Here, we found that breeding macrohabitat selection by Laridae in Cuba could be partly explained through seascape and landscape features of the breeding sites.

Overall, breeding site (macrohabitat) selection by Laridae was mainly explained by lower SST values within 100 and 50 km from colonies. Thus, we show that SST at larger scales played an important role in Cuban Laridae habitat selection, despite its relative seasonal stability throughout tropical waters (Hockey & Wilson, 2003; Jaquemet et al., 2008). The greater contribution of larger spatial scales for SST probably reflects the role of oceanographic conditions (e.g. thermal fronts) at relatively large distances from breeding habitats, coinciding with the foraging range of most species which often exceeds 30 km from colonies (Table S4). Chl *a* at the same spatial scales was also important, although to a lesser extent, for breeding site selection, highlighting the role of marine productivity for breeding. However, it should be noted that Chl *a* is an index of phytoplanktonic biomass that does not match exactly in space with the maximum of forage fish abundance (e.g. Zavalaga et al., 2010).

Sea surface temperature (indirectly) and Chl *a* could be considered as proxies of marine productivity and food availability, and hence key factors for breeding habitat selection by seabirds (Vilchis et al., 2006). However, mismatching patterns between both variables may occur (e.g. Zavalaga et al., 2010). In several regions of the world these variables have been shown as having important effects on seabirds foraging, demography and population dynamics, with generally cooler SST favoring higher Chl *a*, and hence the foraging and breeding success of seabirds (Barbraud et al., 2012; Carroll et al., 2015). The selective pattern of breeding macrohabitat found for Laridae in Cuba is consistent with this general pattern, although the effect of Chl *a* appears lesser than for SST (Figure S1).

The lower contribution of Chl *a* could be consequence of terrestrial runoff leading to eutrophication processes, as occurs in some marine ecosystems of northwestern Cuba (Rey‐Villiers et al., 2021). Thus, such excessive and polluted upwelling of primary productivity may not be attractive for Laridae feeding. This also could be magnified in areas of low marine circulation as in the interior macro‐lagoons (water bodies between the cays and the island of Cuba) of the Sabana‐Camagüey archipelago. In addition, differences in marine current patterns between regions (e.g. Arriaza et al., 2008) and the influence of extreme climate events such as rainfall and hurricanes (Alvarez‐Socorro et al., 2021) should cause differences in local oceanography (e.g. SST, Chl *a*) between coastal areas of Cuba.

Similar to previous studies (e.g. Burger & Gochfeld, 1981, 1986; Greer et al., 1988), areas with moderate to dense vegetation cover were avoided by Laridae for breeding (Figure 4). For seabirds, high cover and dense vegetation cover usually constitutes a barrier to breeding as it limits the visibility and social communication between neighbors at colonies, and hence, may increase predation risk (Bukacinska & Bukacinsky, 1993; Raynor et al., 2012).

### 4.3 Complementarity of CNN and RF approaches

After visual inspection of the image mosaic of 2021, the more suitable predicted areas for Laridae in Cuba included heterogeneous terrestrial covers and waters with contrasting depths. Yet, our ecological understanding here is limited as we have no explicit indication on the metrics CNNs finally used to maximize prediction quality. Some technics exist in order to get insights into the metrics automatically extracted by the CNN, such as layer‐wise backpropagation, saliency maps, “network dissection” and the explainable Artificial Intelligence (xAI) methods (see Borowiec et al., 2022; Pichler & Hartig, 2023; Samek et al., 2021). It is however an active field of research, and using these approaches is beyond the scope of this paper.

Random forests are a classic example of so‐called ensemble models (interacting sets of simple algorithms or statistical models that make up more complex algorithms), which typically have low prediction errors and weight the contribution of the variables analyzed by the model (Pichler & Hartig, 2023). This last property contributes to the explanation of the process or phenomenon being studied. Thus, based on our RFs results and statistical analysis, we could yet hypothesize that important features such as vegetation, number of cays/islets but also ocean color (related to Chl *a*) were captured through Landsat spectral data. Also, the CNN performance might be probably increased including SST information at the radius of 100 km (considering the main results of RFs) and being trained over larger datasets.

This study illustrates the benefits obtained from a complementary analysis of CNN and RF. Here, RF can be seen as an explicative tool relying on features directly related to our a priori ecological hypotheses, while CNN can act as an evaluative tool in order to assess the relevance of habitat spectral features, as well as an efficient predictive tool producing large scale prediction of habitat suitability in a convenient manner. Particularly, CNNs are becoming increasingly important in remote sensing and ecology due to the inclusion of the spatial dimension within their convolutional layers, thus facilitating the identification/characterization of relevant ecological patterns and processes (Hayes et al., 2021). Our prediction of breeding habitat suitability with CNN should also be systematically updated considering the changing dynamics of marine ecosystems and seabird colonies. Finally, we recommend the exploration of building CNNs that use both spectral and relevant ecological data (identified by RF), to produce finer predictions supported ecologically.

### 4.4 Management and conservation implications from the complementary approach

The dispersed distribution pattern of suitable breeding sites in Cuba offers to Laridae a wide variety of options for colony establishment. This could buffer the effects of climate change and anthropogenic pressures on breeding macrohabitats due to presumed vulnerability differences (e.g. flood risk, ease of access to predators and humans, local oceanographic anomalies) among these sites. This also provides flexibility for management agencies, considering the existence of a large number of alternative sites for Laridae conservation in Cuba. Also, the potential for legal protection of some important breeding colonies (e.g. predicted hotspots) is increased due to the low risk of spatial overlap of breeding sites with places of socioeconomic interest, under an appropriate marine spatial planning.

From a practical point of view, we recommend a field validation of the effective presence of colonies in sites that are expected to be highly suitable. This could be done through field surveys at these sites, as a way to optimize logistical and economic resources for conservation purposes. Then, confirmed breeding sites could be considered to update the boundaries of Marine Protected Areas in Cuba, improve the governmental strategy of adaptation to climate change, detect negative effects due to natural and anthropogenic causes (McGowan et al., 2013; Perrow et al., 2015) and set up a sustainable use of marine‐coastal ecosystems (tourism, fishing, industry). Effective conservation measures for seabird populations must necessarily include both the establishment sites of colonies and their surrounding waters (Oppel et al., 2018). More precisely, according to our complementary CNN‐RF approach, conservation and management actions for Laridae breeding macrohabitats in Cuba should include the areas of predicted breeding hotspots. Also, oceanic characteristics (SST and Chl *a*) at mesoscale (50–100 km) around the cays, as well as the degree of dense and moderate vegetation cover, should be considered in future management plans.

## AUTHOR CONTRIBUTIONS

**Antonio Garcia‐Quintas:** Conceptualization (lead); data curation (equal); formal analysis (lead); investigation (equal); methodology (equal); validation (equal); writing – original draft (lead); writing – review and editing (equal). **Amédée Roy:** Formal analysis (equal); methodology (equal); software (lead); validation (lead); visualization (equal); writing – original draft (lead); writing – review and editing (equal). **Christophe Barbraud:** Methodology (equal); supervision (lead); validation (equal); visualization (equal); writing – review and editing (lead). **Hervé Demarcq:** Data curation (lead); methodology (equal); validation (equal); visualization (equal); writing – review and editing (equal). **Dennis Denis:** Conceptualization (equal); formal analysis (equal); methodology (equal); supervision (lead); validation (equal); writing – review and editing (equal). **Sophie Lanco Bertrand:** Conceptualization (equal); formal analysis (equal); funding acquisition (lead); methodology (equal); supervision (lead); validation (equal); visualization (equal); writing – review and editing (lead).

## CONFLICT OF INTEREST STATEMENT

The authors declare no conflicts of interest.

## Supporting information


Figure S1

Table S1

Table S2

Table S3

Table S4

Table S5

Table S6

## Data Availability

The data from this study are available in the Supporting Information (Tables [Link], [Link] and S6). The used satellite images can be downloaded from https://eos.com.
