# Supplementary material for: Machine and deep learning approaches to understand and predict habitat suitability for seabird breeding
Source: Ecol Evol. 2023 Sep 17;13(9):e10549. doi: 10.1002/ece3.10549 (PMC10505760; doi:10.1002/ece3.10549)

**Figure S1** Summaries of general interannually variability of Sea Surface Temperature (SST, first four pages) and Chlorophyll *a* (Chl *a*, last three pages) oceanographic variables in Cuba.

D J F


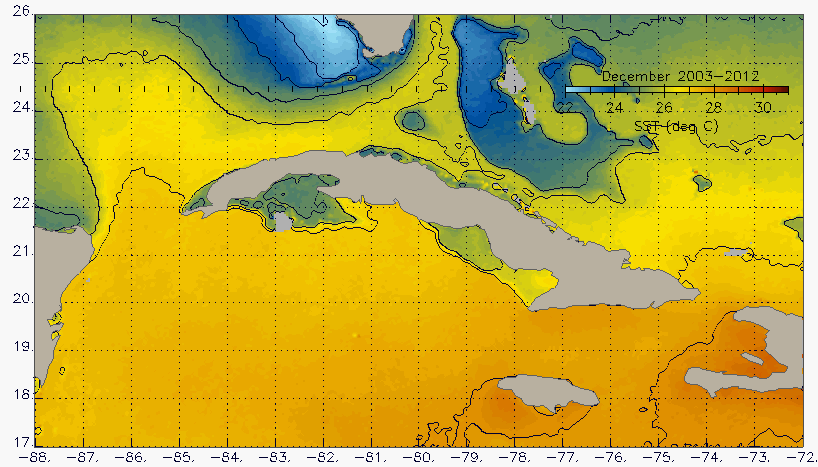


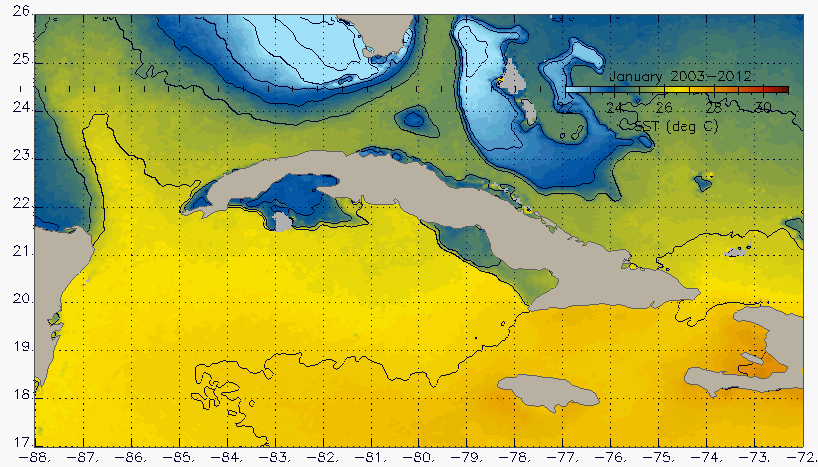


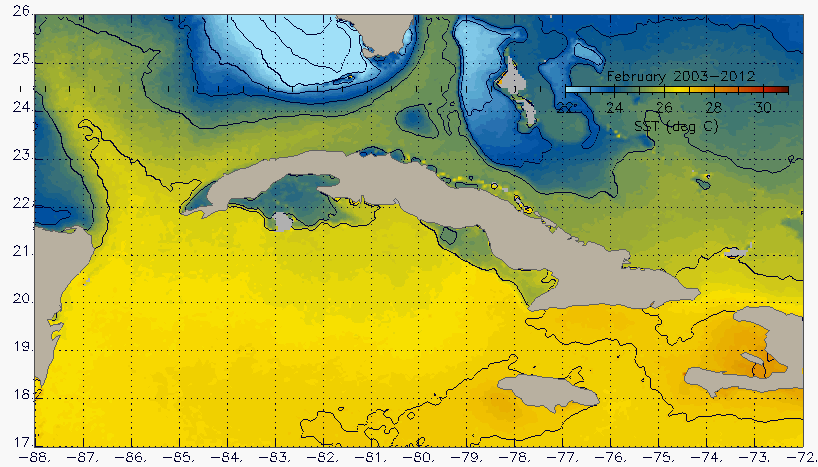


M A M
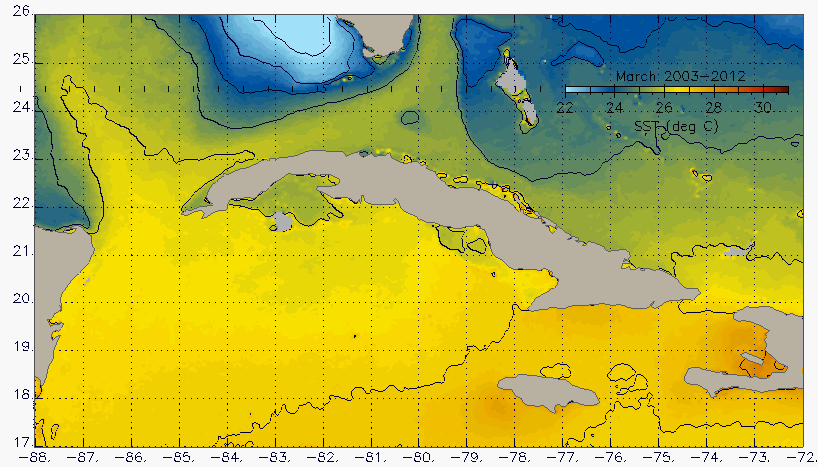

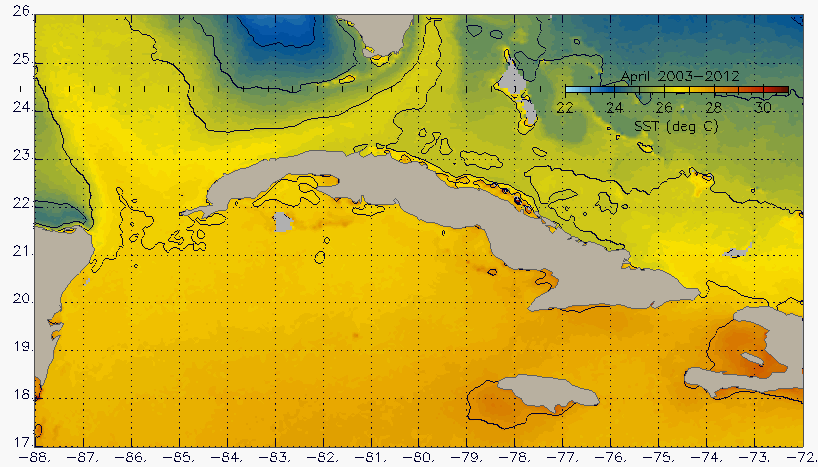

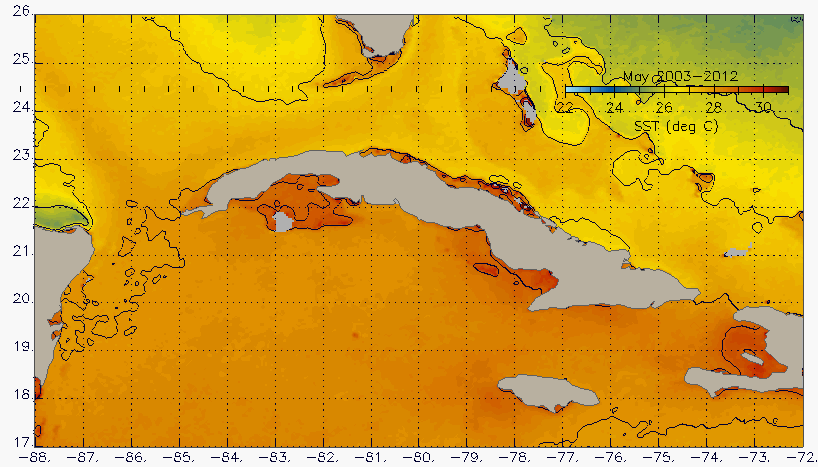


J J A
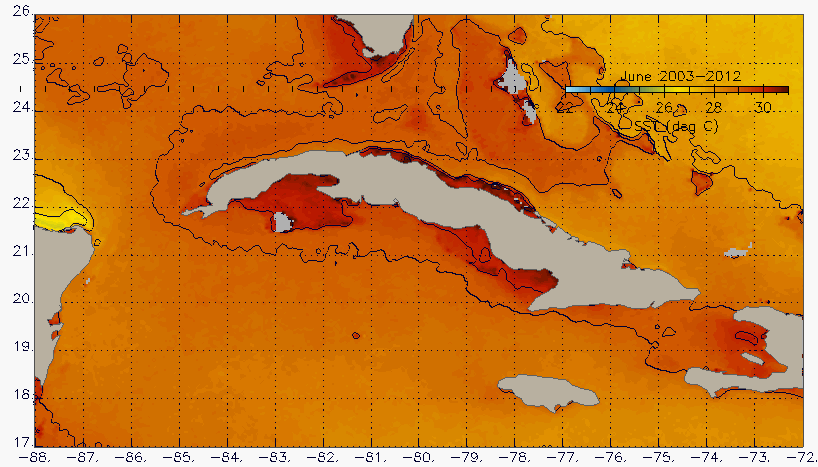

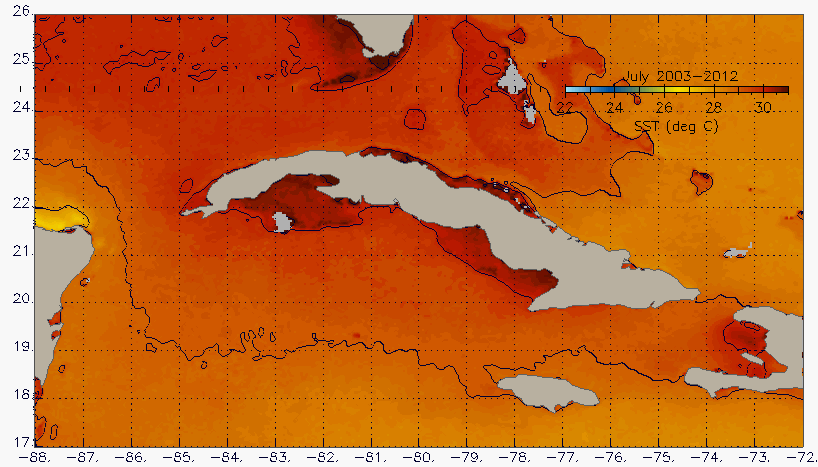

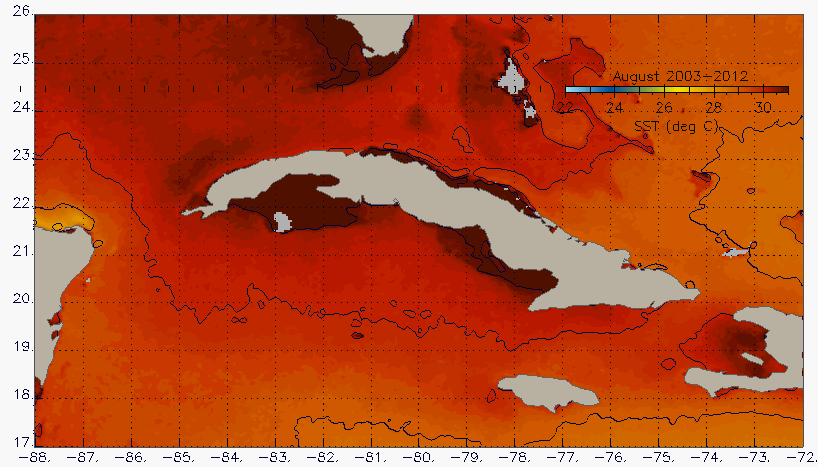


S O N


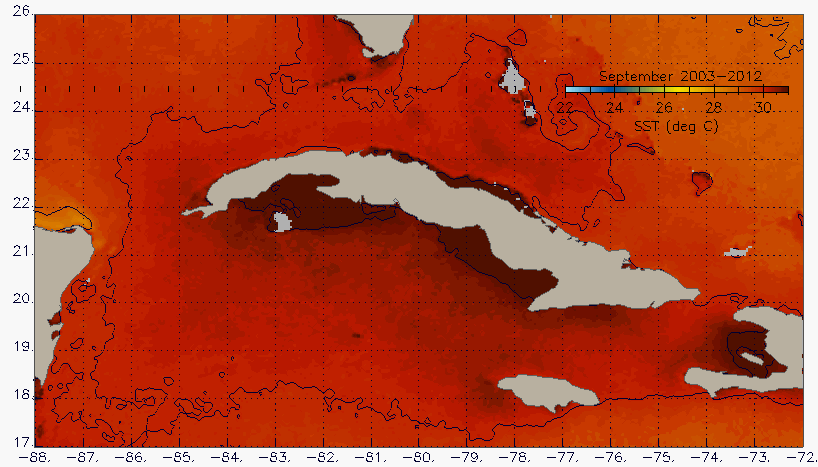


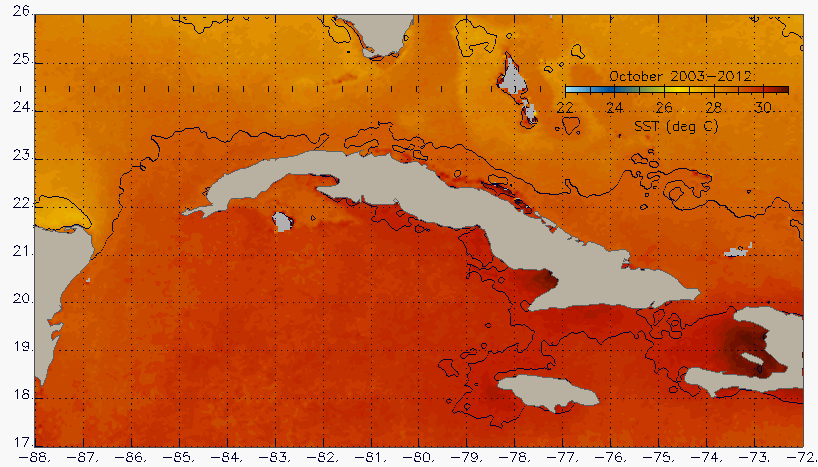


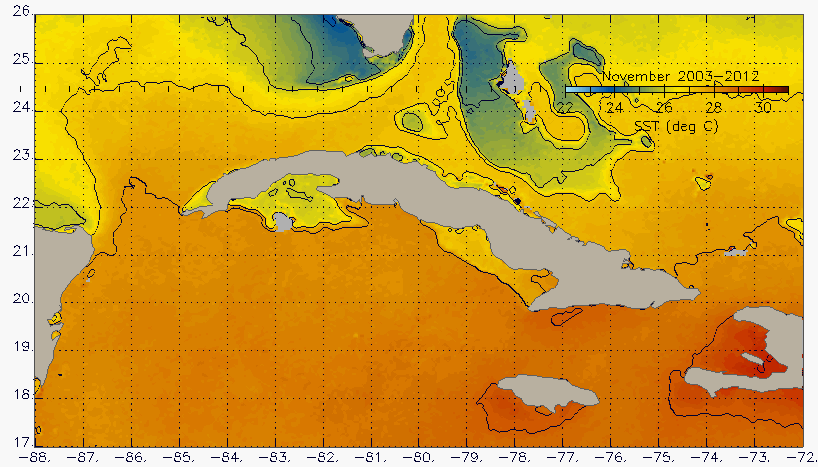


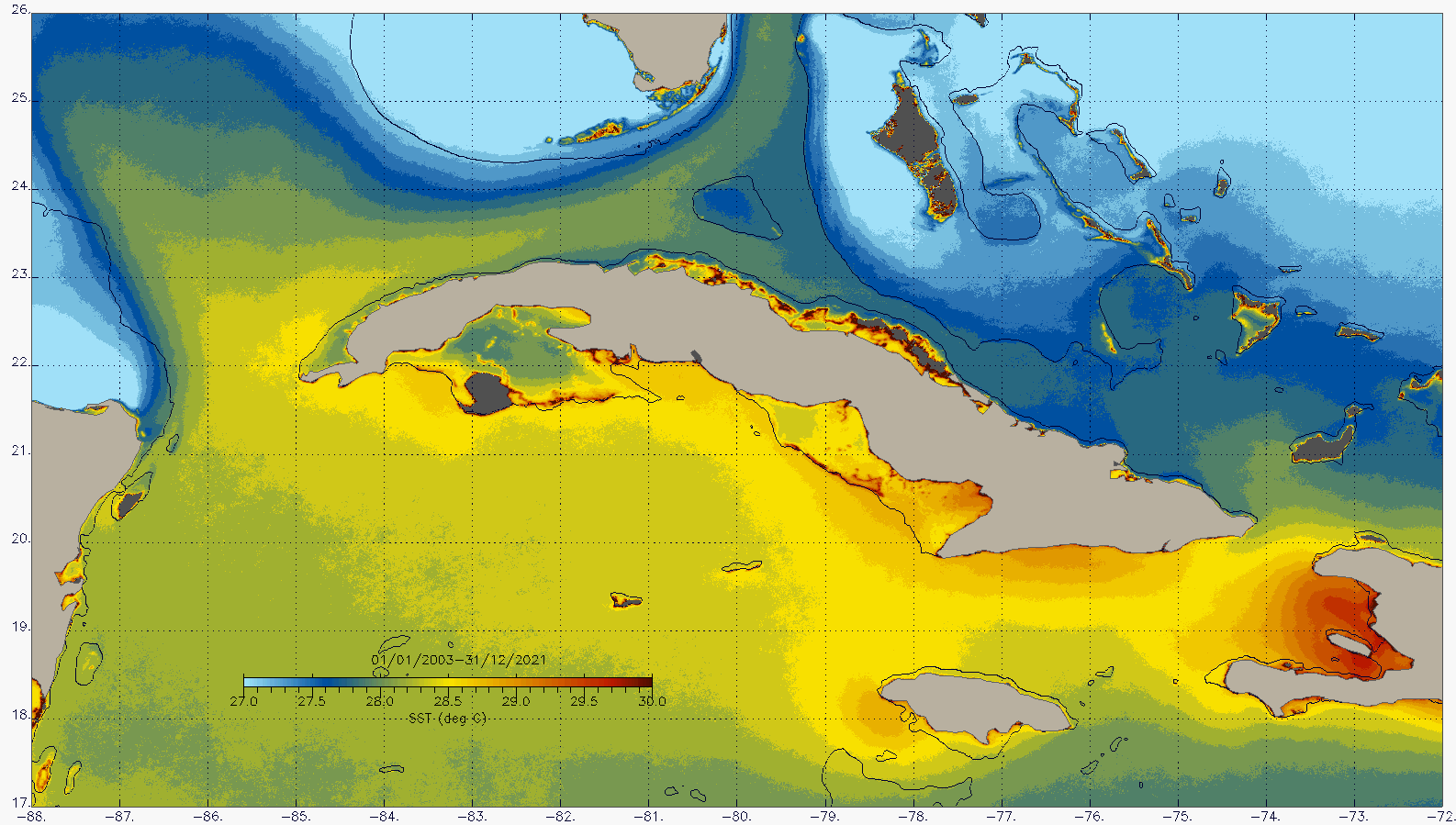


v, 'cuba1_modis_sst2_1km_19y_20030101_20211231.AQUA.R2019.0.qual1.avg.avg.avg.gz', min=27, max=30, xcbo=0.15, ycbo=0.15, /fill, /mar, g_s=1, bathy=200, /png

v, 'cuba1_modis_sst2std_1km_19y_20030101_20211231.AQUA.R2019.0.qual1.avg.avg.sst2.gz', min=0.5, max=3, xcbo=0.15, ycbo=0.15, color=15, /mar, g_s=1, bathy=200, /fil, /png.gz', max=1

Yearly average


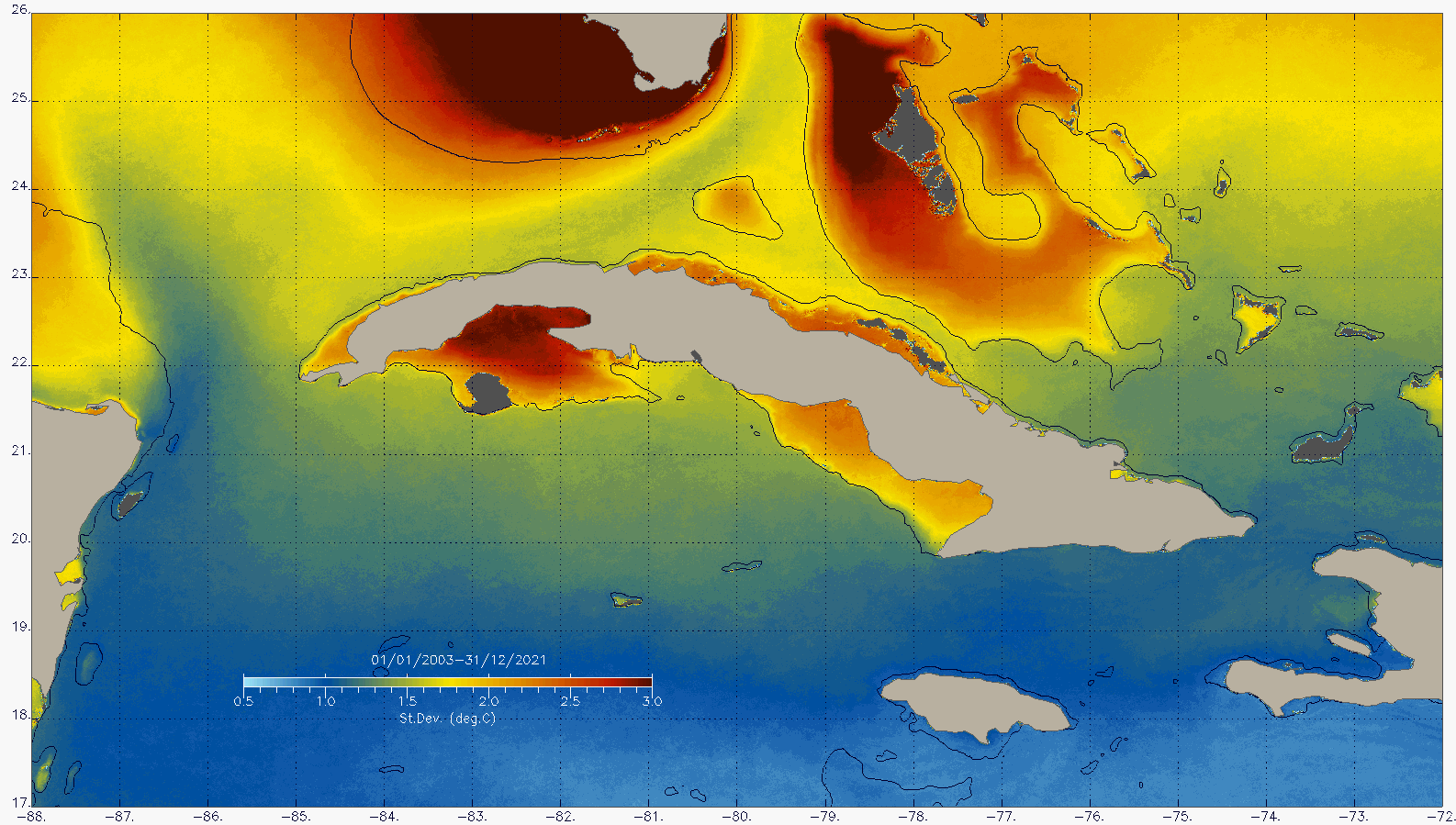


v, 'cuba1_modis_sst2_1km_19y_20030101_20211231.AQUA.R2019.0.qual1.avg.avg.avg.gz', min=27, max=30, /fill, /mar, g_s=1, bathy=200, /png

v, 'cuba1_modis_sst2std_1km_19y_20030101_20211231.AQUA.R2019.0.qual1.avg.avg.sst2.gz', min=0.5, max=3, xcbo=0.15, ycbo=0.15, color=1, /mar, g_s=1, bathy=200, /fil, /png

Seasonal variabilty (St. Dev., scale from 0.5 to 3°C)

(St. Dev. , scale from 0.5 to 3°C)

**Interannual variability**

(seasonally averaged) from 12 months of SSTstd

/data/areas/cuba1/modis/R2019/sst2/1m/bil/

(much lower (about 30%) than the seasonal var.)

avg_cli_month, 'cuba1_modis_sst2_1km_1m_*.AQUA.R2019.0.qual1.avg.gz', /std, 2003, 2021 avg_l, cuba1_modis_sst2std_1km_1m_2003*_2021*.AQUA.R2019.0.qual1.avg.sst2.gz'

v, 'cuba1_modis_sst2std_1km_19y_20030101_20211231.AQUA.R2019.0.qual1.avg.sst2.avg.gz', min=0., max=1, xcbo=0.15, ycbo=0.15, color=0, /mar, g_s=1, bathy=200, /fil, /png


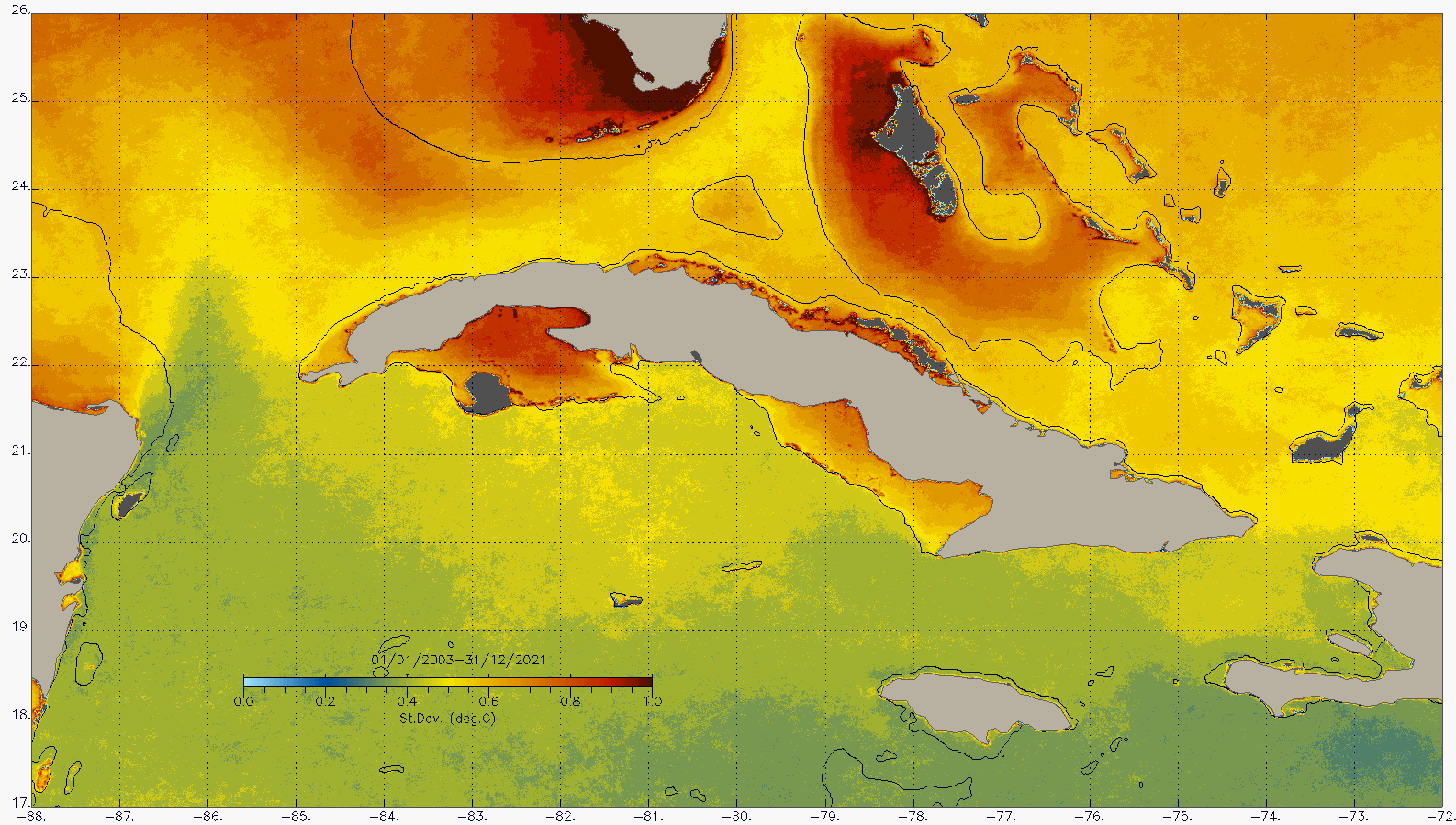


(St. Dev. , scale from 0. to 1°C)

D J F


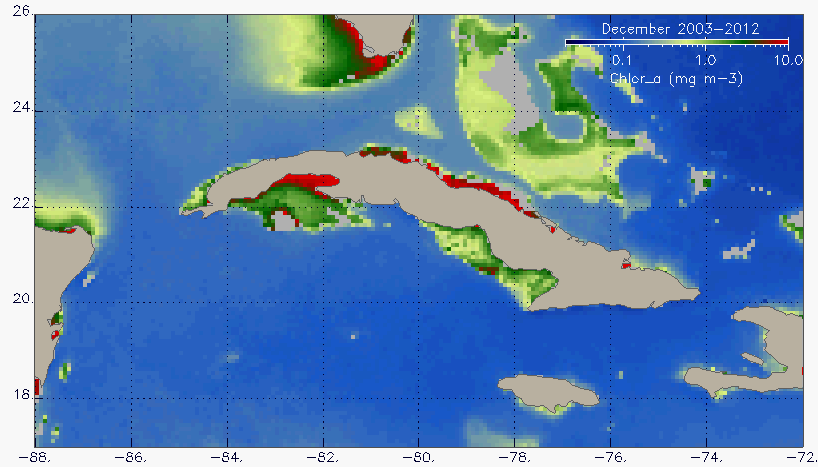


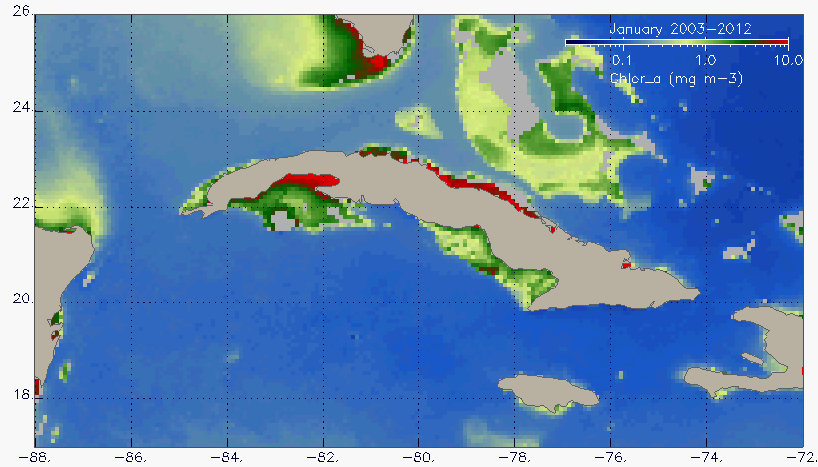


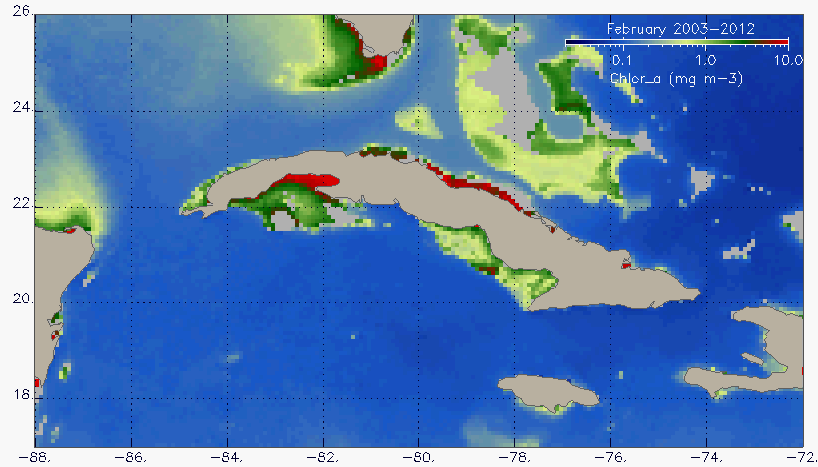


M A M
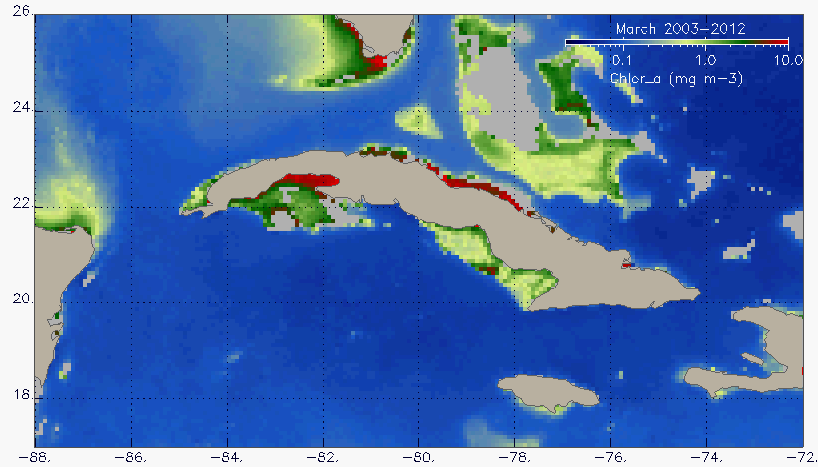

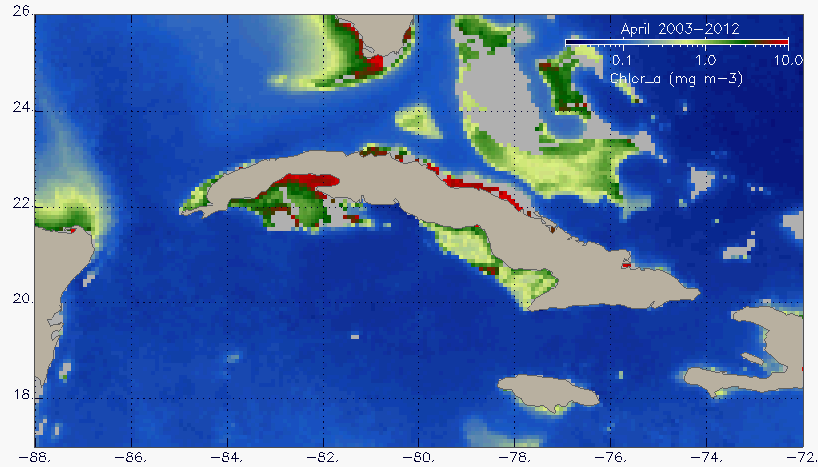

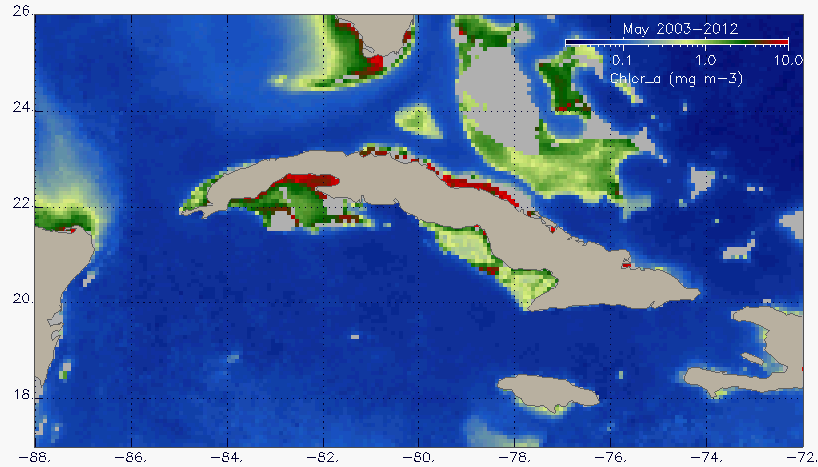


J J A
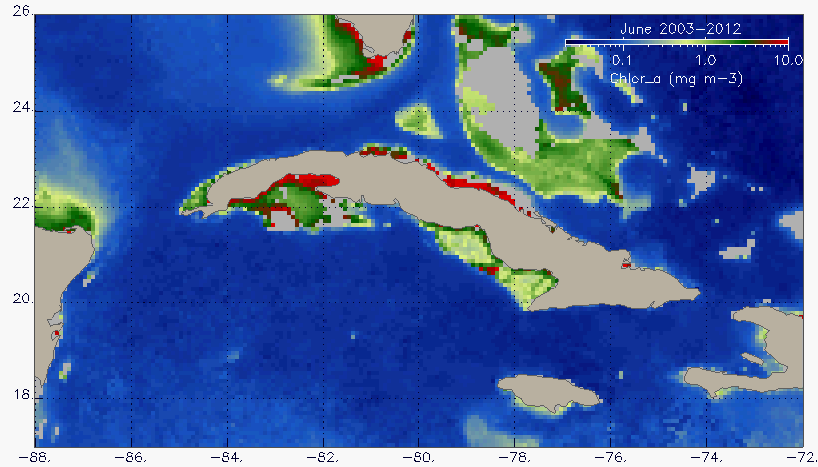

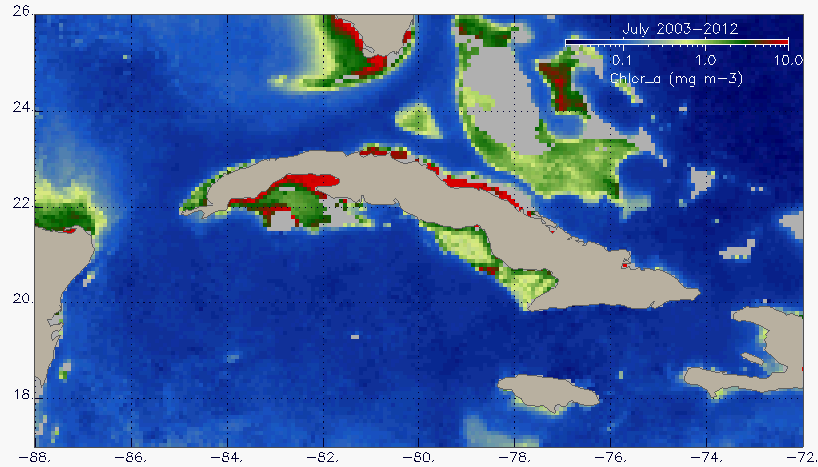

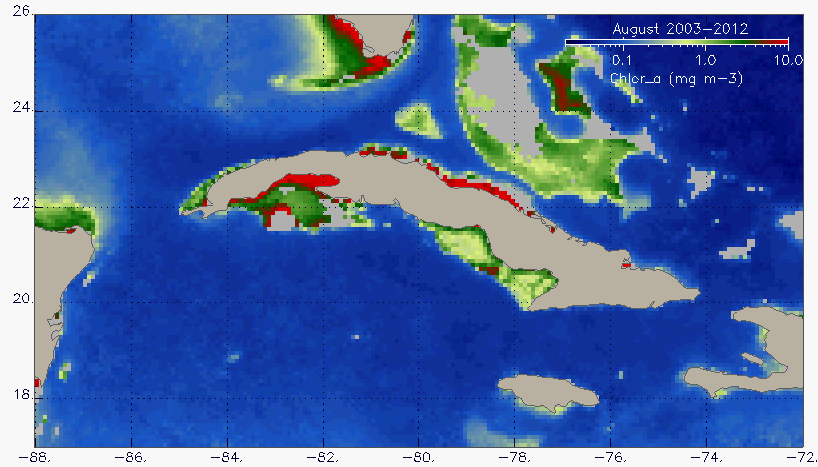


S O N


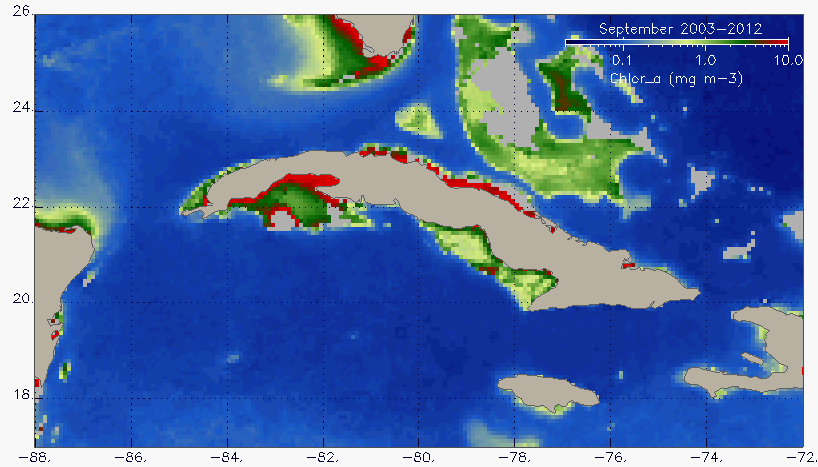


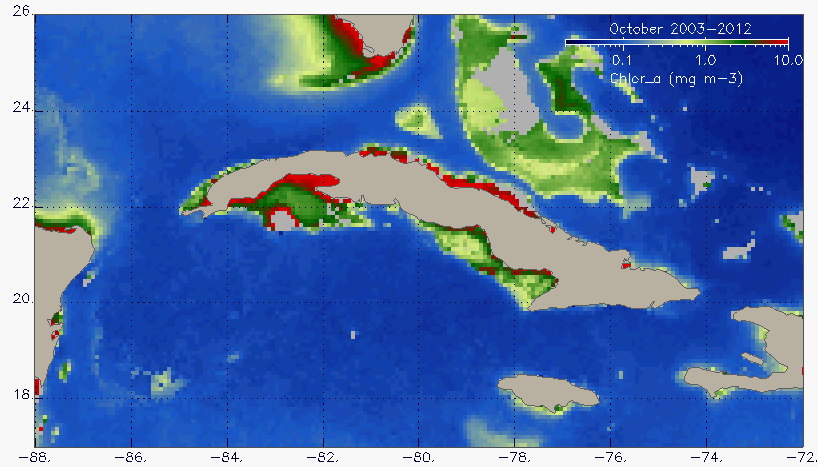


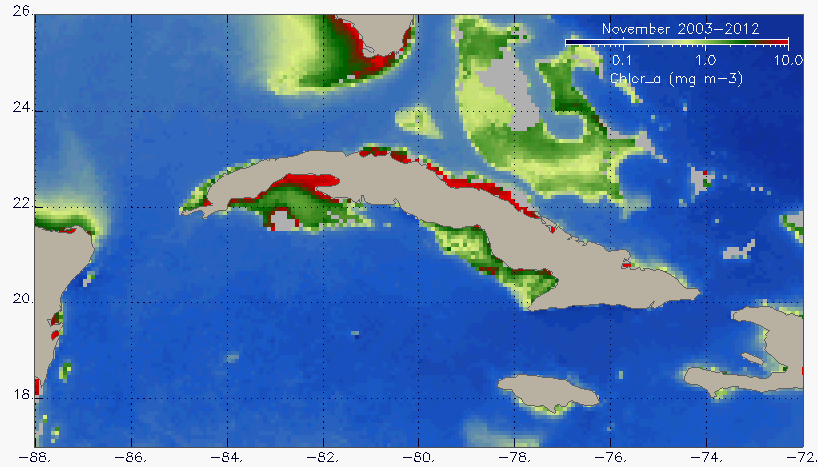


1-km resolution data (area "cuba1")

Checking the variability (seasonal and interannual)

v, 'cuba1_modis_cvar_1km_1m_2003*_2021*.AQUA.R2018.0.avg.chla.gz', /w, max=1, /pix, /png

avg_l, ‘cuba1_modis_chla_1km_1m_20030101_20210131.AQUA.R2018.0.avg.avg.gz’, /cv

v, ‘cuba1_modis_cvar_1km_19y_20030101_20211231.AQUA.R2018.0.avg.avg.chla.gz', max=1

Seasonal variabilty (very low)


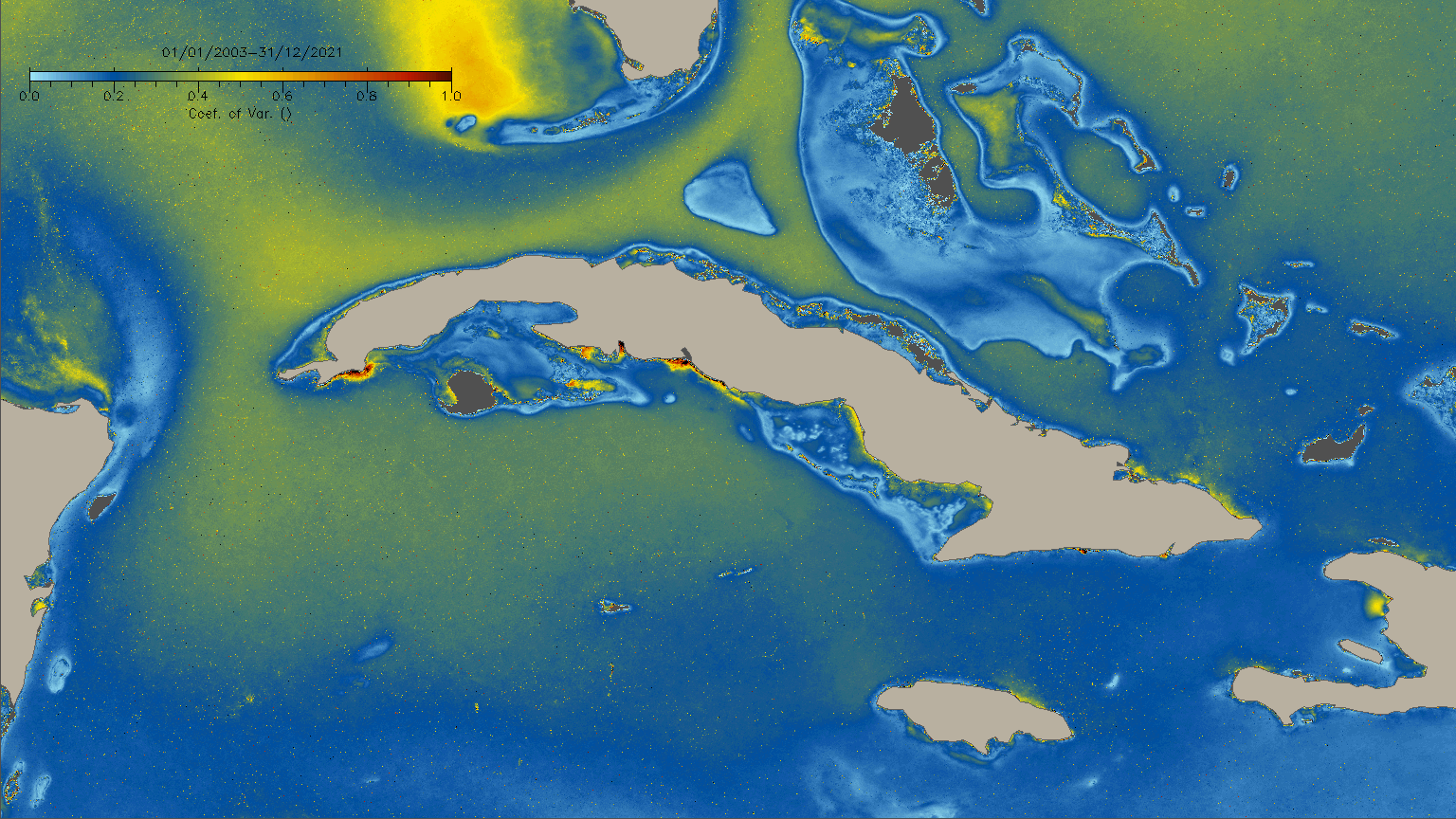


**Interannual variability**

(slightly higher than the seasonal var. but rather low anyway...)

(seasonally averaged) from 12 months cvar cuba1_modis_cvar_1km_1m_2003*_2021*.AQUA.R2018.0.avg.chla.gz


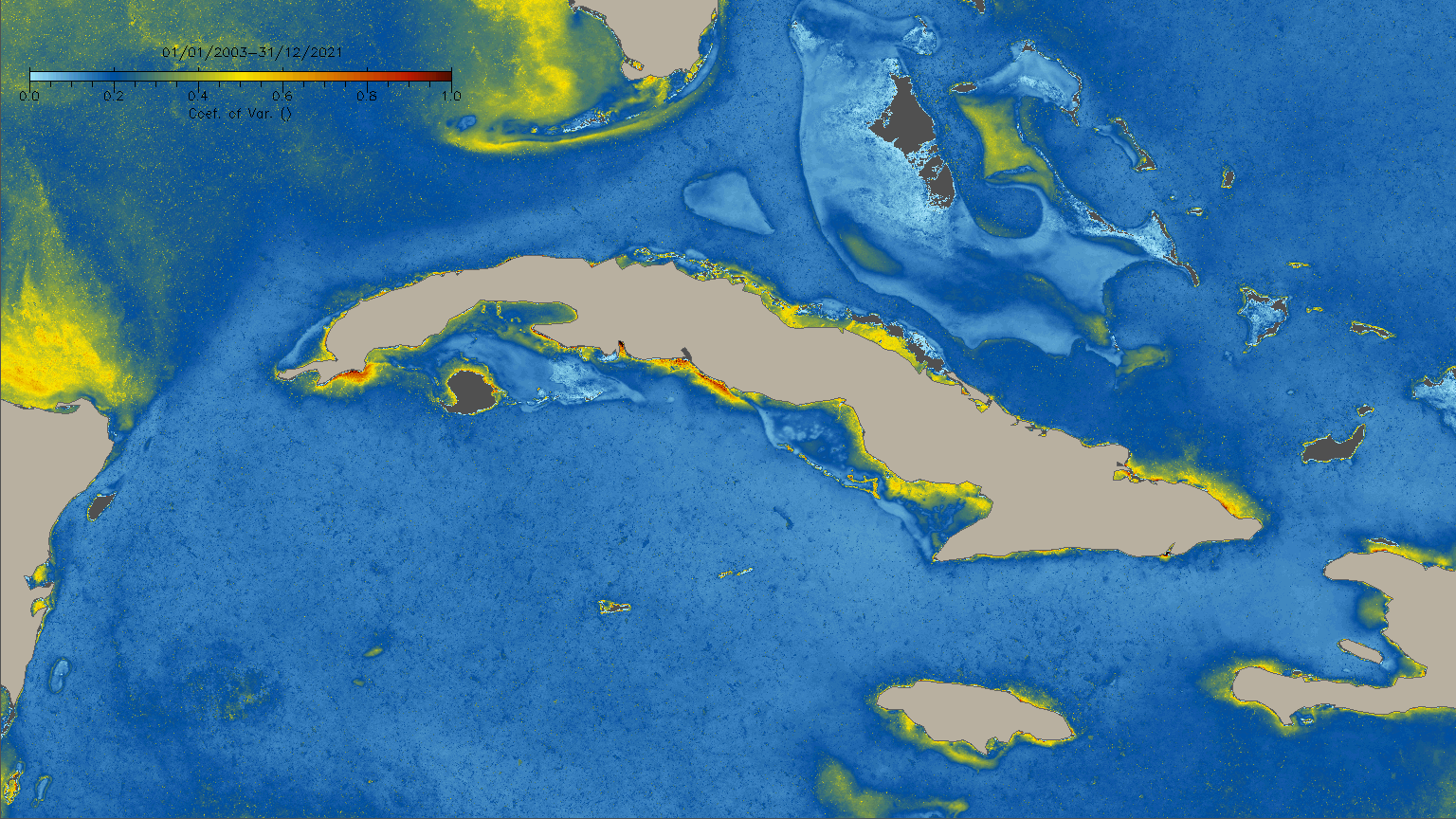

Supplement: Supplementary file 1 — Figure S1 [file ECE3-13-e10549-s001.docx]
