# Supplementary material for: Machine and deep learning approaches to understand and predict habitat suitability for seabird breeding
Source: Ecol Evol. 2023 Sep 17;13(9):e10549. doi: 10.1002/ece3.10549 (PMC10505760; doi:10.1002/ece3.10549)
Supplement: Supplementary file 2 — Table S1 [file ECE3-13-e10549-s007.docx]

**TABLE S1** List of the breeding and available sites of Laridae registered in Cuba. LAGU = laughing gull *Leucophaeus atricilla*, BRNO = brown noddy *Anous stolidus*, SOTE = sooty tern *Onychoprion fuscatus*, BRTE *Onychoprion anaethetus* = bridled tern, LETE = least tern *Sternulla antillarum*, GBTE = gull-billed tern *Gelochelidon nilotica*, ROST = roseate tern *Sterna dougallii*, ROYT = royal tern *Thalasseus maximus*, SATE *Thalasseus sandvicensis* = sandwich tern.

| **Site** | **Longitude** | **Latitude** | **Date of last register** | **Nesting species** | **Source** |
| --- | --- | --- | --- | --- | --- |
| Rincón del Guanal point | -82.83345 | 21.45010 | 1980 | LETE | 2, 7 |
| Borracho cay | -79.15140 | 22.65071 | 1989 | LAGU, BRNO, SOTE | 7 |
| Caimán de Sotavento cay | -78.96420 | 22.69748 | 1989 | LAGU, BRNO, BRTE, ROYT | 7 |
| Monos de Jutías cay | -79.85020 | 22.96936 | 1989 | SOTE, BRTE, LETE | 7 |
| Dios cays | -81.17457 | 21.63407 | 1991 | BRNO, SOTE, BRTE | 7 |
| La Vela cay | -79.75536 | 22.94368 | 1998 | BRNO, BRTE, LETE, ROST, ROYT | 7 |
| Grande cay | -79.21170 | 20.99063 | 1998 | LETE | 12 |
| Tortuga cay | -79.37771 | 21.28699 | 1998 | LAGU, ROYT, SATE | 12 |
| Dune of Bergantines | -78.83738 | 20.93286 | 1998 | LAGU | 7 |
| Hicacos point | -81.14283 | 23.20414 | 2000 | LETE, ROST | 7 |
| Máximo river mouth | -77.46020 | 21.70534 | 2001 | LAGU | 7 |
| Cinco Leguas cay | -80.84182 | 23.12689 | 2002 | LAGU, ROYT | 3 |
| Galindo cay | -80.87131 | 23.25341 | 2002 | LAGU, LETE, ROYT | 3 |
| Palma cay | -79.10301 | 22.38926 | 2002 | ROYT | 7 |
| Jaula cay | -78.51431 | 22.56962 | 2002 | LAGU, BRNO, SOTE, BRTE, ROYT | 9 |
| Cruz cay | -77.77895 | 22.19509 | 2002 | LETE | 3 |
| Fogón cay | -77.73093 | 22.08174 | 2002 | LAGU | 9 |
| South of Guajaba cay | -77.50694 | 21.80528 | 2002 | LAGU, LETE | 9 |
| East of Sifonte cay | -77.41333 | 21.72945 | 2002 | GBTE | 3 |
| Lengua de Pájaro, Sabinal cay | -77.38889 | 21.71979 | 2002 | LAGU, LETE | 1 |
| Nuevitas bay | -77.26694 | 21.49167 | 2002 | LETE | 3 |
| Tío Pepe cay | -79.15389 | 22.62917 | 2003 | LETE | 3 |
| Juan García cay | -83.62580 | 21.97959 | 2003 | LETE | 7 |
| Leonero lagoon | -77.04925 | 20.64129 | 2003 | LAGU, LETE, ROYT | 7 |
| Mono Grande cay | -81.08951 | 23.26596 | 2004 | BRNO, SOTE, BRTE, ROST | 3 |
| Jato inlet | -77.34083 | 21.64556 | 2004 | GBTE | 1, 7 |
| Sijú cay | -83.51313 | 21.94642 | 2004 | LETE | 7 |
| Las Salinas | -81.27723 | 22.11073 | 2004 | LAGU, LETE | 3 |
| Dune Palo Quemado, Fragoso cay | -79.61961 | 22.80563 | 2005 | LETE, ROYT | 7 |
| Fragoso cay | -79.47301 | 22.71754 | 2005 | LETE, ROYT | 7 |
| Marcos cays | -79.65007 | 22.80607 | 2005 | BRTE, SATE | 7 |
| Pajonal cays | -79.68842 | 22.85227 | 2006 | ROST, SATE | 7 |
| Francés point | -83.19415 | 21.63073 | 2006 | LETE, ROST | 8 |
| Español de Afuera cay | -79.13443 | 22.65377 | 2007 | LAGU, SOTE, BRTE, ROYT, SATE | 10 |
| Mono cay | -79.68358 | 22.84578 | 2007 | BRTE, ROST, SATE | 7 |
| Caimán de los Cayuelos cays | -78.90530 | 22.69279 | 2007 | LAGU, BRNO | 10 |
| Caimán de Barlovento cay | -78.87128 | 22.68394 | 2007 | LAGU, BRTE | 10 |
| Caimán cay at Northwest of Caimán de Bella | -78.84632 | 22.67453 | 2007 | BRTE | 10 |
| Caimán de Bella cay | -78.84143 | 22.66959 | 2007 | LETE | 10 |
| Dutton or Pajonal Anegado cay | -79.67746 | 22.89152 | 2008 | BRTE, ROST, SATE | 7 |
| Yuraguanal cay | -77.77126 | 22.11570 | 2013 | ROYT | 4 |
| Verde cay | -77.64759 | 22.11741 | 2013 | LAGU, LETE | 3, 4 |
| Los Ballenatos cay | -81.63857 | 21.57841 | 2013 | LAGU, BRTE, LETE, ROST, ROYT, SATE | 3, 7 |
| Loma del Puerto beach, Coco cay | -78.42420 | 22.55401 | 2014 | LETE | 5 |
| Pescador cay | -83.74932 | 22.80580 | 2017 | LETE | 11 |
| Inés de Soto cay | -83.77728 | 22.81612 | 2019 | LETE | 11 |
| Felipe de Sotavento cay | -78.64486 | 22.62660 | 2021 | LAGU, BRNO, SOTE, BRTE | 5, 9 |
| Felipe de Barlovento cay | -78.62352 | 22.61220 | 2021 | LAGU, BRNO, SOTE, BRTE, ROST, ROYT, SATE | 6, 9 |
| Paredón de Lado cay | -78.21316 | 22.47886 | 2021 | LAGU, BRNO, SOTE, BRTE, ROYT | 5, 9 |
| Available 1 | -81.14300 | 23.20400 | 2020 | - | Fieldwork |
| Available 2 | -79.18600 | 22.59600 | 2020 | - | Fieldwork |
| Available 3 | -78.75800 | 22.38400 | 2020 | - | Fieldwork |
| Available 4 | -78.68900 | 22.62700 | 2020 | - | Fieldwork |
| Available 5 | -78.62500 | 22.61400 | 2020 | - | Fieldwork |
| Available 6 | -78.33100 | 22.52500 | 2020 | - | Fieldwork |
| Available 7 | -78.35700 | 22.52300 | 2020 | - | Fieldwork |
| Available 8 | -78.17000 | 22.48200 | 2020 | - | Fieldwork |
| Available 9 | -78.14800 | 22.48300 | 2020 | - | Fieldwork |
| Available 10 | -78.14600 | 22.44000 | 2020 | - | Fieldwork |
| Available 11 | -78.08900 | 22.42600 | 2020 | - | Fieldwork |
| Available 12 | -77.87100 | 22.21100 | 2020 | - | Fieldwork |
| Available 13 | -77.74400 | 22.12900 | 2020 | - | Fieldwork |
| Available 14 | -77.32700 | 21.73100 | 2020 | - | Fieldwork |
| Available 15 | -77.14000 | 21.661 | 2020 | - | Fieldwork |
| Available 16 | -76.31000 | 21.25100 | 2020 | - | Fieldwork |
| Available 17 | -77.02600 | 20.54400 | 2020 | - | Fieldwork |
| Available 18 | -75.86300 | 20.00000 | 2020 | - | Fieldwork |
| Available 19 | -78.43000 | 20.63600 | 2020 | - | Fieldwork |
| Available 20 | -78.55400 | 20.69400 | 2020 | - | Fieldwork |
| Available 21 | -78.71800 | 20.66600 | 2020 | - | Fieldwork |
| Available 22 | -78.75100 | 20.67800 | 2020 | - | Fieldwork |
| Available 23 | -78.94500 | 20.80700 | 2020 | - | Fieldwork |
| Available 24 | -78.88400 | 20.80400 | 2020 | - | Fieldwork |
| Available 25 | -78.75900 | 20.78800 | 2020 | - | Fieldwork |
| Available 26 | -78.96500 | 20.87000 | 2020 | - | Fieldwork |
| Available 27 | -78.93200 | 20.97900 | 2020 | - | Fieldwork |
| Available 28 | -78.95400 | 21.07500 | 2020 | - | Fieldwork |
| Available 29 | -78.80400 | 21.39400 | 2020 | - | Fieldwork |
| Available 30 | -78.73700 | 21.53700 | 2020 | - | Fieldwork |
| Available 31 | -78.88700 | 21.53400 | 2020 | - | Fieldwork |
| Available 32 | -7866700 | 22.22600 | 2020 | - | Fieldwork |
| Available 33 | -78.32600 | 22.24400 | 2020 | - | Fieldwork |
| Available 34 | -79.35200 | 21.08100 | 2020 | - | Fieldwork |
| Available 35 | -79.45700 | 21.12800 | 2020 | - | Fieldwork |
| Available 36 | -79.19000 | 21.01400 | 2020 | - | Fieldwork |
| Available 37 | -79.97400 | 21.72800 | 2020 | - | Fieldwork |
| Available 38 | -80.42100 | 22.07700 | 2020 | - | Fieldwork |
| Available 39 | -79.14800 | 22.40000 | 2020 | - | Fieldwork |
| Available 40 | -77.28300 | 20.69400 | 2020 | - | Fieldwork |
| Available 41 | -78.58300 | 22.51800 | 2020 | - | Fieldwork |
| Available 42 | -77.91900 | 22.31300 | 2020 | - | Fieldwork |
| Available 43 | -77.84400 | 22.16100 | 2020 | - | Fieldwork |
| Available 44 | -77.21600 | 21.52100 | 2020 | - | Fieldwork |
| Available 45 | -78.96700 | 22.66500 | 2020 | - | Fieldwork |
| Available 46 | -78.54000 | 22.29400 | 2020 | - | Fieldwork |
| Available 47 | -77.90500 | 22.25400 | 2020 | - | Fieldwork |
| Available 48 | -77.43600 | 20.61500 | 2020 | - | Fieldwork |
| Available 49 | -79.04200 | 21.20100 | 2020 | - | Fieldwork |
| Available 50 | -79.19900 | 21.17900 | 2020 | - | Fieldwork |
| Available 51 | -83.65500 | 22.83600 | 2020 | - | Fieldwork |
| Available 52 | -83.70500 | 22.80700 | 2020 | - | Fieldwork |

^1^Barrio, O., Soriano, R. & Paneca, G. (2001). Colonias de nidificación de aves acuáticas en Cayo Sabinal. *Informe Proyecto Sabana-Camagüey CUB/98/G32*. Centro de Investigaciones de Medio Ambiente de Camagüey, 6 pp.

^2^Berovides, V., & Smith, R. (1983). Aspectos ecológicos de la nificación de *Sterna hirundo* y *Sterna albifrons*. *Ciencias Biológicas, 9*, 128-131.

^3^Blanco, P. (2006). Distribución y áreas de importancia para las aves del orden Charadriiformes en Cuba. *Tesis de Doctorado*. Universidad de La Habana, Cuba. Pp: 87.

^4^Figueredo-Martín, T., Hernández-Fernández, L., García-Quintas, A. & Marichal, E. (2013). Solicitud de Licencia Ambiental a Sweet Spa: “Actividad de Pesca Recreativa en Cayo Cruz”. *Informe de servicio científico-técnico*. Centro de Investigaciones de Ecosistemas Costeros, Cuba.

^5^García-Quintas, A. (pers.com.). Ornithologist, Centro de Investigaciones de Ecosistemas Costeros, Cuba.

^6^García-Quintas, A., González, L. & González, A. (2020). Novedades sobre la reproducción de dos especies de aves marinas poco comunes en Cuba. *Journal of Caribbean Ornithology,* *33*, 54-57.

^7^ Jiménez, A., Rodríguez, P., & Blanco, P. (2009). Cuba. In P. Bradley & R. Norton (Eds.), *An inventory of breeding seabirds of the Caribbean* (pp. 47-57). University Press of Florida.

^8^Rodríguez, A., Rodríguez, P. & Zayas, R. (2009). Nidificación de la Gaviotica (Sternula antillarum) y la Gaviota Común (*Sterna hirundo*) en el sur de la Isla de la Juventud, Cuba. *Journal of Caribbean Ornithology,* *22*, 96-97.

^9^ Rodríguez, P., Rodríguez, D., Pérez, E., Llanes, A., Blanco, P., Barrio, O., Parada, A., Ruiz, E., Socarrás, E., Hernández, A., & Cejas, F. (2003). Distribución y composición de las colonias de nidificación de aves acuáticas en el Archipiélago de Sabana-Camagüey. *CD-ROM Memorias del VII Simposio de Botánica*. ISBN: 959-270-029-X3.

^10^Ruiz, E., Arias, A., Más, L., Romero, M., García, J. M. & Arias, R. (2010). Breve caracterización de las colonias reproductivas de aves acuáticas (Charadriiformes) presentes en los cayos del noreste de Villa Clara, Cuba. *ECOVIDA, 2(2)*, 57-68.

^11^Ruiz, I. (pers.com.). Conservation specialist of the Los Colorados protected area, National Company for the Flora and Fauna Conservation, Cuba.

^12^Socarrás, E., Parada, A., López, M., Gómez, R. & Aguilar, A. (2006). Biota terrestre del ecosistema Jardines de la Reina. In Ecosistemas costeros: biodiversidad y gestión de recursos naturales. Compilación por el XV Aniversario del CIEC. Sección II. Ecosistema Jardines de la Reina. CIEC. Editorial CUJAE. ISBN: 959-261-254-4.
