# Supplementary material for: Machine and deep learning approaches to understand and predict habitat suitability for seabird breeding
Source: Ecol Evol. 2023 Sep 17;13(9):e10549. doi: 10.1002/ece3.10549 (PMC10505760; doi:10.1002/ece3.10549)
Supplement: Supplementary file 3 — Table S2 [file ECE3-13-e10549-s005.docx]

**TABLE S2** List of satellite images used to characterize the Laridae breeding habitat and predict its suitability in Cuba. A = derived images since Landsat 5 -7 and Sentinel-2 satellites, B = derived images since MODIS-Aqua satellite that cover a general scene of Cuba.

A)

| **Satellite** | **Scene** | **Date acquired (yy-mm-dd)** |
| --- | --- | --- |
| Landsat 5 | 14/44 | 1989-09-19 |
| Landsat 5 | 15/45 | 1991-04-09 |
| Landsat 5 | 15/45 | 1991-09-16 |
| Landsat 5 | 13/46 | 1998-05-16 |
| Landsat 5 | 13/46 | 1998-06-01 |
| Landsat 5 | 13/46 | 1998-07-19 |
| Landsat 5 | 13/46 | 1998-08-20 |
| Landsat 5 | 14/44 | 1998-05-07 |
| Landsat 5 | 14/44 | 1998-06-08 |
| Landsat 5 | 14/44 | 1998-07-10 |
| Landsat 5 | 14/44 | 1998-08-27 |
| Landsat 5 | 14/45 | 1998-05-07 |
| Landsat 5 | 14/45 | 1998-06-08 |
| Landsat 5 | 14/45 | 1998-07-10 |
| Landsat 5 | 14/45 | 1998-08-11 |
| Landsat 5 | 14/45 | 2000-05-03 |
| Landsat 5 | 14/45 | 2000-06-20 |
| Landsat 7 | 15/44 | 2000-07-30 |
| Landsat 7 | 15/44 | 2000-08-15 |
| Landsat 7 | 12/45 | 2001-05-25 |
| Landsat 7 | 12/45 | 2001-06-10 |
| Landsat 7 | 12/45 | 2001-07-12 |
| Landsat 7 | 12/45 | 2001-08-29 |
| Landsat 7 | 12/45 | 2002-04-26 |
| Landsat 7 | 12/45 | 2002-06-29 |
| Landsat 7 | 12/45 | 2002-07-15 |
| Landsat 7 | 13/44 | 2002-04-17 |
| Landsat 7 | 13/44 | 2002-07-22 |
| Landsat 7 | 13/44 | 2002-08-23 |
| Landsat 7 | 13/45 | 2002-04-17 |
| Landsat 7 | 13/45 | 2002-06-04 |
| Landsat 7 | 13/45 | 2002-08-23 |
| Landsat 7 | 14/44 | 2002-05-10 |
| Landsat 7 | 14/44 | 2002-06-27 |
| Landsat 7 | 14/44 | 2002-07-29 |
| Landsat 7 | 14/44 | 2002-08-30 |
| Landsat 7 | 15/44 | 2002-05-17 |
| Landsat 7 | 15/44 | 2002-07-04 |
| Landsat 7 | 15/44 | 2002-08-05 |
| Landsat 7 | 12/46 | 2003-05-15 |
| Landsat 7 | 12/46 | 2003-08-03 |
| Landsat 7 | 14/44 | 2003-05-13 |
| Landsat 5 | 14/44 | 2003-07-08 |
| Landsat 7 | 14/44 | 2003-08-17 |
| Landsat 7 | 16/45 | 2003-04-25 |
| Landsat 7 | 16/45 | 2003-08-15 |
| Landsat 7 | 12/45 | 2004-05-17 |
| Landsat 7 | 12/45 | 2004-06-18 |
| Landsat 7 | 12/45 | 2004-07-20 |
| Landsat 7 | 12/45 | 2004-08-21 |
| Landsat 5 | 15/44 | 2004-05-30 |
| Landsat 7 | 15/44 | 2004-06-07 |
| Landsat 7 | 15/44 | 2004-08-10 |
| Landsat 7 | 15/45 | 2004-05-22 |
| Landsat 7 | 15/45 | 2004-06-07 |
| Landsat 7 | 15/45 | 2004-08-10 |
| Landsat 7 | 16/45 | 2004-05-13 |
| Landsat 7 | 16/45 | 2004-08-17 |
| Landsat 7 | 14/44 | 2005-05-02 |
| Landsat 5 | 14/44 | 2005-08-14 |
| Landsat 7 | 14/44 | 2006-05-05 |
| Landsat 7 | 14/44 | 2006-06-22 |
| Landsat 7 | 14/44 | 2006-07-08 |
| Landsat 7 | 14/44 | 2006-08-25 |
| Landsat 7 | 16/45 | 2006-05-19 |
| Landsat 7 | 16/45 | 2006-06-04 |
| Landsat 7 | 16/45 | 2006-07-22 |
| Landsat 7 | 16/45 | 2006-08-23 |
| Landsat 7 | 13/44 | 2007-05-01 |
| Landsat 7 | 13/44 | 2007-06-18 |
| Landsat 7 | 13/44 | 2007-09-06 |
| Landsat 7 | 14/44 | 2007-05-08 |
| Landsat 7 | 14/44 | 2007-06-09 |
| Landsat 7 | 14/44 | 2007-09-13 |
| Landsat 7 | 14/44 | 2008-05-26 |
| Landsat 7 | 14/44 | 2008-08-14 |
| Landsat 7 | 13/45 | 2013-05-01 |
| Landsat 7 | 13/45 | 2013-07-20 |
| Landsat 7 | 13/45 | 2013-09-06 |
| Landsat 7 | 15/45 | 2013-05-15 |
| Landsat 7 | 15/45 | 2013-08-03 |
| Landsat 7 | 13/44 | 2014-05-20 |
| Landsat 7 | 13/44 | 2014-06-21 |
| Landsat 7 | 13/44 | 2014-07-23 |
| Landsat 7 | 13/44 | 2014-08-08 |
| Landsat 7 | 10-46 | 2015-05-18 |
| Landsat 7 | 10-46 | 2015-08-22 |
| Landsat 7 | 11/46 | 2020-05-06 |
| Landsat 7 | 11/46 | 2020-06-23 |
| Landsat 7 | 11/46 | 2020-07-09 |
| Landsat 7 | 11/46 | 2020-08-10 |
| Landsat 7 | 12/45 | 2020-05-29 |
| Landsat 7 | 12/45 | 2020-06-30 |
| Landsat 7 | 12/45 | 2020-07-16 |
| Landsat 7 | 12/45 | 2020-08-17 |
| Landsat 7 | 12/46 | 2020-05-13 |
| Landsat 7 | 12/46 | 2020-06-30 |
| Landsat 7 | 12/46 | 2020-07-16 |
| Landsat 7 | 12/46 | 2020-08-17 |
| Landsat 7 | 13/44 | 2020-05-04 |
| Landsat 7 | 13/44 | 2020-06-05 |
| Landsat 7 | 13/44 | 2020-07-07 |
| Landsat 7 | 13/44 | 2020-08-08 |
| Landsat 7 | 13/45 | 2020-05-04 |
| Landsat 7 | 13/45 | 2020-06-05 |
| Landsat 7 | 13/45 | 2020-07-07 |
| Landsat 7 | 13/45 | 2020-08-08 |
| Landsat 7 | 13/46 | 2020-05-04 |
| Landsat 7 | 13/46 | 2020-06-05 |
| Landsat 7 | 13/46 | 2020-07-23 |
| Landsat 7 | 13/46 | 2020-08-08 |
| Landsat 7 | 14/44 | 2020-05-27 |
| Landsat 7 | 14/44 | 2020-06-28 |
| Landsat 7 | 14/44 | 2020-07-14 |
| Landsat 7 | 14/44 | 2020-08-31 |
| Landsat 7 | 14/45 | 2020-05-27 |
| Landsat 7 | 14/45 | 2020-06-28 |
| Landsat 7 | 14/45 | 2020-07-14 |
| Landsat 7 | 14/45 | 2020-08-31 |
| Landsat 7 | 15/44 | 2020-05-02 |
| Landsat 7 | 15/44 | 2020-08-22 |
| Landsat 7 | 17/44 | 2020-05-16 |
| Landsat 7 | 17/44 | 2020-06-01 |
| Landsat 7 | 17/44 | 2020-07-03 |
| Landsat 7 | 11-45 | 2021-07-12 |
| Landsat 7 | 11-45 | 2021-08-29 |
| Landsat 7 | 11-46 | 2021-06-04 |
| Landsat 7 | 11-46 | 2021-07-06 |
| Landsat 7 | 11-46 | 2021-08-29 |
| Landsat 7 | 12-45 | 2021-05-16 |
| Landsat 7 | 12-45 | 2021-07-03 |
| Landsat 7 | 12-46 | 2021-07-03 |
| Landsat 7 | 13-44 | 2021-05-07 |
| Landsat 7 | 13-44 | 2021-06-08 |
| Landsat 7 | 13-44 | 2021-07-26 |
| Landsat 7 | 13-45 | 2021-05-07 |
| Landsat 7 | 13-45 | 2021-06-24 |
| Landsat 7 | 13-46 | 2021-05-23 |
| Landsat 7 | 13-46 | 2021-06-24 |
| Landsat 7 | 13-46 | 2021-07-10 |
| Landsat 7 | 14-44 | 2021-05-30 |
| Landsat 7 | 14-44 | 2021-07-17 |
| Landsat 7 | 14-45 | 2021-05-14 |
| Landsat 7 | 14-45 | 2021-07-17 |
| Landsat 7 | 15-44 | 2021-07-08 |
| Landsat 7 | 15-44 | 2021-08-25 |
| Landsat 7 | 15-45 | 2021-07-24 |
| Landsat 7 | 15-45 | 2021-08-25 |
| Landsat 7 | 16-44 | 2021-07-31 |
| Landsat 7 | 16-44 | 2021-09-01 |
| Landsat 7 | 16-45 | 2021-05-28 |
| Landsat 7 | 16-45 | 2021-09-01 |
| Landsat 7 | 17-44 | 2021-08-07 |
| Landsat 7 | 17-45 | 2021-08-23 |
| Sentinel-2 | # 17QQF | 2021-05-27 |
| Sentinel-2 | # 17QQE | 2021-06-01 |

B)

| **Starting date (mm-dd)** | **Ending date (mm-dd)** | **Year** |
| --- | --- | --- |
| 07-04 | 07-29 | 2002 |
| 08-07 | 08-31 | 2002 |
| 05-01 | 08-31 | 2003 |
| 05-01 | 08-31 | 2004 |
| 05-01 | 08-31 | 2005 |
| 05-01 | 08-31 | 2006 |
| 05-01 | 08-31 | 2007 |
| 05-01 | 08-31 | 2008 |
| 05-01 | 08-31 | 2009 |
| 05-01 | 08-31 | 2010 |
| 05-01 | 08-31 | 2011 |
| 05-01 | 08-31 | 2012 |
| 05-01 | 08-31 | 2013 |
| 05-01 | 08-31 | 2014 |
| 05-01 | 08-31 | 2015 |
| 05-01 | 08-31 | 2016 |
| 05-01 | 08-31 | 2017 |
| 05-01 | 08-31 | 2018 |
| 05-01 | 08-31 | 2019 |
| 05-01 | 08-31 | 2020 |
| 05-01 | 08-31 | 2021 |
