# Supplementary material for: Machine and deep learning approaches to understand and predict habitat suitability for seabird breeding
Source: Ecol Evol. 2023 Sep 17;13(9):e10549. doi: 10.1002/ece3.10549 (PMC10505760; doi:10.1002/ece3.10549)
Supplement: Supplementary file 4 — Table S3 [file ECE3-13-e10549-s006.docx]

| **Datasets** | **Images by habitat classes (breeding vs available)** | **Training sample size (70% of data)** | **Validation**  **sample size (30% of data)** | **Dense layers** | **Units** | **Epochs** | **Validation**  **accuracy (by image)** | **Validation**  **F1-score (by image)** | **Validation**  **accuracy (by locality)** | **Validation**  **F1-score (by locality)** |
| --- | --- | --- | --- | --- | --- | --- | --- | --- | --- | --- |
| Images with any cloud cover | 149 vs 208 | 254 | 103 | 5 | 156 | 25 | 75.7 | 76.2 | 86.7 | 85.7 |
| Images with cloud cover < 30% | 132 vs 176 | 216 | 92 | 5 | 512 | 25 | 72.8 | 73.7 | 86.7 | 84.6 |

**TABLE S3** Characteristics of two variants of a dataset (since six channels of Landsat images) and best architectures (dense layers, processing units and epochs) of convolutional neural networks models used to classify breeding localities of Laridae in Cuba. Performance indicators (accuracy and F1-score) are expressed in %. Channels used were red, green, blue, near infrared, shortwave infrared 1 and shortwave infrared 2.
