# Supplementary material for: Machine and deep learning approaches to understand and predict habitat suitability for seabird breeding
Source: Ecol Evol. 2023 Sep 17;13(9):e10549. doi: 10.1002/ece3.10549 (PMC10505760; doi:10.1002/ece3.10549)
Supplement: Supplementary file 5 — Table S4 [file ECE3-13-e10549-s002.docx]

**TABLE S4** Maximum foraging ranges of nine Laridae species during the breeding season at different world localities and its estimated values for the Cuban archipelago.

| **Species** | **Foraging habitat^a^** | **Mean maximum foraging range (km)** | **Estimated maximum foraging range in Cuba (km)** | **Supporting explanation** |
| --- | --- | --- | --- | --- |
| Laughing gull *Leucophaeus atricilla* | Nearshore | 45.0^14^  30.0^16^  >40.0^20^ | 45.0 | This species has large reproductive colonies (up to 250 pairs) in Cuba despite the fact that the collected information corresponds to foraging in open oceans. |
| Brown noddy (*Anous stolidus*) | Between Nearshore and Offshore | 24.0 and 80.5^4^  ≈180.0^15^  54.9^21^  269.0^24^ | 55.0 | Selected value that correspond to the foraging localities at seas/gulfs (similar to the Cuban context); moreover two references belong to a geographic area near to Cuba (Caribbean Sea and the northeast of the Gulf of Mexico). |
| Sooty tern (*Onychoprion fuscatus*) | Offshore | 420.0-1130.0 and 50.0-130.0^4^  450.0^8^  480.0-600.0^15^  895.0^23^  128.0^24^ | 100.0 | Selected value that correspond to the foraging localities at seas/gulfs (similar to Cuban context); moreover two references belong to a geographic area near to Cuba (Caribbean Sea and the NE of the Gulf of Mexico). |
| Bridled tern (*Onychoprion anaethetus*) | Nearshore and Offshore | 28.0^5^  2.5^6^  30.0^9^  80.0^18^ | 30.0 | Selected value that correspond to the foraging localities at seas/gulfs (similar to the Cuban context). |
| Least tern (*Sternula antillarum*) | Nearshore | 4.9^1^  <5.0^2^  5.0 and 8.0^4^  1.5^10^  12.0^12^ | 8.0 | Selected value that correspond to the foraging localities at seas/gulfs (similar to the Cuban context); reference that shows the value = 12 km belongs to a scenery (salt flat in Oklahoma, USA) less frequent in Cuba. |
| Gull-billed tern (*Gelochelidon nilotica*) | Nearshore | <5.0^2^  ≈45.0^3^  45.0^4^ | 45.0 | Selected value that correspond to the foraging localities at seas/gulfs (similar to the Cuban context). |
| Roseate tern (*Sterna dougallii*) | Nearshore | 5.0 and 22.0^4^  60.0^15^  30.0^19^ | 22.0 | Selected value that correspond to the foraging localities at seas/gulfs (similar to the Cuban context). |
| Royal tern (*Thalasseus maximus*) | Nearshore | 65.0^4^  50.0^11^  55.0^13^  30.0^16^  158.0^24^  ≈42.0^25^ | 55.0 | Selected value because all compiled data correspond to the foraging localities in open oceans (tendency to major ranges); 158 km is an atypical value derived of 13 sampled individuals in Senegal (median = 36); value=65 km was resulted of inaccurate methods; moreover, the colonies in Cuba are not large (<90 pairs). |
| Sandwich tern (*Thalasseus sandvicensis*) | Nearshore | 25.0^4^  16.0^7^  31.0^13^  30.0^16^  54.0^17^  54.0^19^  61.0^22^  ≈42.0^25^ | 54.0 | Selected value that correspond to the foraging localities at seas/gulfs (similar to the Cuban context); Thaxter *et al*. (2012) show summary values of world´s compilations; 61 km is an exceptional value when the central tendency statistics are analyzed (in Fijn *et al*., 2017). |

^1^Tomkins, I. R. (1959). Life History Notes on the Least Tern. *The Wilson Bulletin*, *71*(4), 313–322.

^2^Erwin, R. M. (1978). Coloniality in terns: The role of social feeding. *The Condor*, *80*(2), 211. https://doi.org/10.2307/1367920

^3^Møller, A. P. (1982). Coloniality and colony structure in Gull-billed Terns Gelochelidon nilotica. *Journal für Ornithologie*, *123*(1), 41-53.

^4^Clapp, R.B., D. Morgan-Jacobs, & Banks, R.C. (1983). Marine birds of the Southeastern United States. US Fish and Wildlife Service, FWS/OBS 83/30, Washington, DC.

^5^Hulsman, K. (1984). Survey of seabird colonies in the Capricornia Section of the Great Barrier Reef Marine Park 111. Population parameters and management strategies. *Research Report to Great Barrier Reef Marine Park Authority*.

^6^Hulsman, K., & Langham, N. P. E. (1985). Breeding biology of the Bridled Tern Sterna anaethetus. *Emu*, *85*(4), 240-249.

^7^Fasola, M., & Bogliani, G. (1990). Foraging ranges of an assemblage of Mediterranean seabirds. *Colonial Waterbirds*, *13*(1), 72–74.

^8^Flint, E. N. (1991). Time and energy limits to the foraging radius of Sooty Terns Sterna fuscata. *Ibis*, *133*(1), 43–46. https://doi.org/10.1111/j.1474-919X.1991.tb04808.x

^9^Kohno, H., & Kishimoto, H. (1991). Prey of the Bridled Tern Sterna anaethetus on Nakanokamishima Island, South Ryukyus, Japan. *Japanese Journal of Ornithology*, *40*(1), 15–25. https://doi.org/10.3838/jjo.40.15

^10^Wilson, E. C., Hubert, W. A., & Anderson, S. H. (1993). Nesting and foraging of Least Terns on Sand Pits in Central Nebraska. *The Southwestern Naturalist*, *38*(1), 9. https://doi.org/10.2307/3671637

^11^Lee, D. S. (1995). Marine birds off the coast of North Carolina. *The Chat, 59*, 113-188.

^12^Schweitzer, S. H., & Leslie, D. M. (1996). Foraging patterns of the Least Tern (Sterna antillarum) in North-Central Oklahoma. *The Southwestern Naturalist*, *41*(3), 307–314.

^13^McGinnis, T. W., & Emslie, S. D. (2001). The Foraging Ecology of Royal and Sandwich Terns in North Carolina, USA. *Waterbirds: The International Journal of Waterbird Biology*, *24*(3), 361. https://doi.org/10.2307/1522066

^14^Dosch, J. J. (2003). Movement Patterns of Adult Laughing Gulls Larus atricilla During the Nesting Season. *Acta Ornithologica*, *38*(1), 15–25. https://doi.org/10.3161/068.038.0106

^15^Surman, C. A., & Wooller, R. D. (2003). Comparative foraging ecology of five sympatric terns at a sub-tropical island in the eastern Indian Ocean. *J. Zool., Lond.*, *259*, 219–230.

^16^Wickliffe, L. C., & Jodice, P. G. R. (2010). *Seabird attendance at shrimp trawlers in nearshore waters of South Carolina*. 10.

^17^Perrow, M. R., Skeate, E. R., & Gilroy, J. J. (2011). Visual tracking from a rigid-hulled inflatable boat to determine foraging movements of breeding terns: Visual Tracking of Terns. *Journal of Field Ornithology*, *82*(1), 68–79. https://doi.org/10.1111/j.1557-9263.2010.00309.x

^18^Dunlop, J. N., & Surman, C. A. (2012). The role of foraging ecology in the contrasting responses of two dark terns to a changing ocean climate. *Marine Ornithology*, *40*, 105–110.

^19^Thaxter, C. B., Lascelles, B., Sugar, K., Cook, A. S. C. P., Roos, S., Bolton, M., Langston, R. H. W., & Burton, N. H. K. (2012). Seabird foraging ranges as a preliminary tool for identifying candidate Marine Protected Areas. *Biological Conservation*, *156*, 53–61. https://doi.org/10.1016/j.biocon.2011.12.009

^20^Washburn, B. E., Bernhardt, G. E., Kutschbach-Brohl, L., Chipman, R. B., & Francoeur, L. C. (2013). Foraging ecology of four gull species at a coastal–urban interface. *The Condor*, *115*(1), 67–76.

^21^Maxwell, S. M., Conners, M. G., Sisson, N. B., & Dawson, T. M. (2016). Potential Benefits and Shortcomings of Marine Protected Areas for Small Seabirds Revealed Using Miniature Tags. *Frontiers in Marine Science*, *3*. https://doi.org/10.3389/fmars.2016.00264

^22^Fijn, R. C., de Jong, J., Courtens, W., Verstraete, H., Stienen, E. W. M., & Poot, M. J. M. (2017). GPS-tracking and colony observations reveal variation in offshore habitat use and foraging ecology of breeding Sandwich Terns. *Journal of Sea Research*, *127*, 203–211. https://doi.org/10.1016/j.seares.2016.11.005

^23^Neumann, J. L., Larose, C. S., Brodin, G., & Feare, C. J. (2018). Foraging ranges of incubating Sooty Terns Onychoprion fuscatus on Bird island, Seychelles, during a transition from food plenty to scarcity, as revealed by gps loggers. *Marine Ornithology*, *46*, 11–18.

^24^Oppel, S., Bolton, M., Carneiro, A. P. B., Dias, M. P., Green, J. A., Masello, J. F., Phillips, R. A., Owen, E., Quillfeldt, P., Beard, A., Bertrand, S., Blackburn, J., Boersma, P. D., Borges, A., Broderick, A. C., Catry, P., Cleasby, I., Clingham, E., Creuwels, J., … Croxall, J. (2018). Spatial scales of marine conservation management for breeding seabirds. *Marine Policy*, *98*, 37–46. https://doi.org/10.1016/j.marpol.2018.08.024

^25^Gatto, A., Yorio, P., Doldan, M. S., & Gomila, L. V. (2019). Spatial and temporal foraging movement patterns in Royal Terns (Thalasseus maximus) and Cayenne Terns (Thalasseus sandvicensis eurygnathus) in Northern Patagonia, Argentina. *Waterbirds*, *42*(2), 217. https://doi.org/10.1675/063.042.0209
