# Supplementary material for: Machine and deep learning approaches to understand and predict habitat suitability for seabird breeding
Source: Ecol Evol. 2023 Sep 17;13(9):e10549. doi: 10.1002/ece3.10549 (PMC10505760; doi:10.1002/ece3.10549)
Supplement: Supplementary file 6 — Table S5 [file ECE3-13-e10549-s003.docx]

**TABLE S5** Overview of the most frequently used performance indicators (derived since confusion matrix) for classification purposes. TP = true positives, TN = true negatives, FP = false positives, FN = false negatives. Indicators values vary between 0 to 1 and can be expressed in %.

| **Indicators** | **Formula** | **Description** |
| --- | --- | --- |
| Overall Accuracy | TP + TN / (TP + TN + FP + FN) | Ratio of true predictions (positive and negative) and the total number of observations. |
| Precision | TP / (TP + FP) | Ratio of true presences classified correctly and the number of all positive predictions. Assesses how many of the predicted presences are actually true. |
| Recall | TP / (TP + FN) | Ratio of true presences classified correctly and the total number of instances belonging to the classes (true positive and false negative). Assess how many of the actual presences were classified as true. |
| F1-score | 2 x (precision x recall) / (precision + recall) | Harmonic mean of recall and precision. Constitute a balanced accuracy metric that is sensitive to both under- and overestimation. |
