# Supplementary material for: Machine and deep learning approaches to understand and predict habitat suitability for seabird breeding
Source: Ecol Evol. 2023 Sep 17;13(9):e10549. doi: 10.1002/ece3.10549 (PMC10505760; doi:10.1002/ece3.10549)
Supplement: Supplementary file 7 — Table S6 [file ECE3-13-e10549-s004.docx]

**TABLE S6** Dataset of the potentially important variables for Laridae breeding macrohabitat selection (breeding site, i.e. cay, islet or coastal site) in Cuba.

| **Sites** | **Spatial scale (km)** | **Area (km^2^)** | **Perimeter (km)** | **Shape index** | **Minimum distance to cay (km)** | **Minimum distance to main island (km)** | **Number of cays** | **Non-flooding zone cover (%)** | **Vegetal cover (%)** | **Vegetal cover_moderate+dense_ (%)** | **SST (°C)** | **Minimum distance to 200-m isobath (km)** | **Chl *a* (mg m^-3^)** |
| --- | --- | --- | --- | --- | --- | --- | --- | --- | --- | --- | --- | --- | --- |
| Breeding | 10 | 0.001 | 0.01 | 0.08 | 0.46 | 0.49 | 4 | 58.33 | 66.67 | 33.33 | 30.44 | 22.07 | 11.65 |
| Breeding | 10 | 0.095 | 2.60 | 2.39 | 0.06 | 27.76 | 84 | 89.52 | 82.86 | 39.05 | 30.06 | 4.07 | 1.16 |
| Breeding | 10 | 0.126 | 2.82 | 2.24 | 0.84 | 33.16 | 51 | 94.29 | 71.43 | 3.57 | 29.56 | 6.53 | 1.09 |
| Breeding | 10 | 8.040 | 118.54 | 11.80 | 0.09 | 5.50 | 72 | 99.38 | 92.54 | 76.37 | 31.26 | 16.75 | 9.91 |
| Breeding | 10 | 0.014 | 0.60 | 1.41 | 4.43 | 29.63 | 2 | 87.50 | 37.50 | 37.50 | 29.70 | 3.51 | 0.68 |
| Breeding | 10 | 0.047 | 1.72 | 2.24 | 0.08 | 29.20 | 47 | 96.15 | 78.85 | 40.38 | 30.12 | 2.67 | 0.74 |
| Breeding | 10 | 0.042 | 1.26 | 1.73 | 2.23 | 29.54 | 64 | 95.74 | 89.36 | 57.45 | 30.09 | 2.02 | 0.64 |
| Breeding | 10 | 0.019 | 0.83 | 1.70 | 0.39 | 22.23 | 34 | 90.48 | 85.71 | 61.90 | 30.24 | 11.28 | 1.32 |
| Breeding | 10 | 4.801 | 56.88 | 7.32 | 0.00 | 18.00 | 51 | 98.52 | 97.50 | 89.79 | 30.61 | 4.52 | 3.75 |
| Breeding | 10 | 0.059 | 1.72 | 2.01 | 0.24 | 6.09 | 20 | 92.31 | 78.46 | 49.23 | 30.31 | 7.37 | 2.62 |
| Breeding | 10 | 0.029 | 0.78 | 1.30 | 3.57 | 8.16 | 13 | 87.50 | 43.75 | 0.00 | 29.02 | 2.93 | 1.61 |
| Breeding | 10 | 0.023 | 1.24 | 2.29 | 2.37 | 33.46 | 50 | 76.92 | 92.31 | 3.85 | 29.94 | 4.03 | 0.61 |
| Breeding | 10 | 0.006 | 0.60 | 2.13 | 0.10 | 2.21 | 29 | 100.00 | 14.29 | 0.00 | 30.29 | 11.09 | 7.28 |
| Breeding | 10 | 0.038 | 0.86 | 1.24 | 0.15 | 5.97 | 25 | 79.69 | 66.67 | 9.38 | 30.36 | 8.11 | 3.46 |
| Breeding | 10 | 16.437 | 90.48 | 6.30 | 0.08 | 25.52 | 108 | 100.00 | 91.73 | 72.26 | 30.38 | 6.38 | 1.42 |
| Breeding | 10 | 0.026 | 0.84 | 1.47 | 6.01 | 30.53 | 9 | 100.00 | 86.21 | 51.72 | 28.82 | 2.28 | 0.64 |
| Breeding | 10 | 0.055 | 1.13 | 1.36 | 0.16 | 16.24 | 32 | 100.00 | 75.41 | 26.23 | 29.75 | 1.10 | 2.18 |
| Breeding | 10 | 0.083 | 1.56 | 1.53 | 0.13 | 4.67 | 19 | 94.57 | 97.83 | 86.96 | 30.10 | 7.58 | 6.66 |
| Breeding | 10 | 0.152 | 0.14 | 0.10 | 0.03 | 8.49 | 30 | 87.71 | 94.08 | 82.25 | 30.06 | 5.77 | 6.09 |
| Breeding | 10 | 0.134 | 0.91 | 0.70 | 0.10 | 6.24 | 14 | 75.84 | 26.85 | 6.71 | 29.92 | 5.65 | 5.13 |
| Breeding | 10 | 1.740 | 14.22 | 3.04 | 0.02 | 1.67 | 15 | 98.28 | 82.76 | 44.25 | 30.56 | 13.01 | 7.35 |
| Breeding | 10 | 14.350 | 44.15 | 3.29 | 0.03 | 0.00 | 8 | 91.44 | 81.95 | 49.76 | 29.63 | 6.60 | 2.91 |
| Breeding | 10 | 0.017 | 0.69 | 1.49 | 0.12 | 32.19 | 7 | 100.00 | 63.16 | 0.00 | 29.55 | 7.61 | 0.99 |
| Breeding | 10 | 1.632 | 8.43 | 1.86 | 0.08 | 12.88 | 18 | 98.63 | 97.08 | 91.23 | 29.97 | 3.24 | 3.04 |
| Breeding | 10 | 0.033 | 1.04 | 1.61 | 0.04 | 13.32 | 32 | 100.00 | 59.46 | 2.70 | 29.56 | 1.17 | 4.18 |
| Breeding | 10 | 7.362 | 27.81 | 2.89 | 0.00 | 6.74 | 31 | 98.75 | 97.83 | 91.44 | 29.73 | 5.42 | 4.66 |
| Breeding | 10 | 0.169 | 2.55 | 1.75 | 4.23 | 0.13 | 8 | 99.88 | 81.91 | 22.34 | 31.23 | 33.43 | 18.07 |
| Breeding | 10 | 0.002 | 0.24 | 1.60 | 3.91 | 37.83 | 5 | 100.00 | 50.00 | 0.00 | 30.30 | 19.26 | 0.76 |
| Breeding | 10 | 0.122 | 1.00 | 0.81 | 0.01 | 7.26 | 51 | 100.00 | 88.89 | 37.78 | 30.30 | 7.74 | 3.33 |
| Breeding | 10 | 60.550 | 36.03 | 1.31 | 0.40 | 1.30 | 33 | 98.65 | 97.08 | 86.97 | 31.68 | 81.13 | 6.96 |
| Breeding | 10 | 0.207 | 5.27 | 3.27 | 0.06 | 45.85 | 6 | 100.00 | 67.83 | 23.48 | 29.85 | 2.03 | 0.69 |
| Breeding | 10 | 23.370 | 193.17 | 11.27 | 0.00 | 58.49 | 2 | 96.53 | 92.38 | 68.16 | 30.09 | 3.01 | 0.71 |
| Breeding | 10 | 21.950 | 135.90 | 8.18 | 1.83 | 28.36 | 25 | 100.00 | 76.81 | 33.30 | 30.02 | 7.94 | 0.98 |
| Breeding | 10 | 2.224 | 25.80 | 4.88 | 0.02 | 24.65 | 3 | 96.24 | 91.46 | 40.64 | 30.26 | 3.42 | 1.86 |
| Breeding | 10 | 0.011 | 0.79 | 2.14 | 0.68 | 0.00 | 62 | 100.00 | 8.33 | 0.00 | 30.99 | 9.70 | 1.80 |
| Breeding | 10 | 9.073 | 47.72 | 4.47 | 0.01 | 19.16 | 1 | 99.64 | 93.50 | 72.22 | 30.35 | 2.98 | 1.79 |
| Breeding | 10 | 70.950 | 240.67 | 8.06 | 0.00 | 3.74 | 40 | 97.82 | 92.45 | 67.64 | 30.28 | 6.81 | 4.45 |
| Breeding | 10 | 0.155 | 0.61 | 0.44 | 0.04 | 0.47 | 1 | 87.79 | 90.70 | 73.26 | 29.95 | 1.89 | 2.03 |
| Breeding | 10 | 0.004 | 0.29 | 1.36 | 0.37 | 65.98 | 34 | 100.00 | 25.00 | 0.00 | 29.86 | 1.64 | 0.30 |
| Breeding | 10 | 0.014 | 0.95 | 2.31 | 1.13 | 28.18 | 81 | 100.00 | 40.00 | 0.00 | 30.14 | 4.07 | 1.18 |
| Breeding | 10 | 0.064 | 2.71 | 3.02 | 0.05 | 31.35 | 7 | 100.00 | 49.30 | 0.00 | 29.48 | 6.90 | 1.01 |
| Breeding | 10 | 0.005 | 0.56 | 2.36 | 0.17 | 30.93 | 5 | 100.00 | 0.00 | 0.00 | 29.48 | 6.57 | 1.02 |
| Breeding | 10 | 0.253 | 4.29 | 2.41 | 0.17 | 30.29 | 5 | 99.01 | 84.32 | 5.35 | 29.48 | 6.71 | 1.02 |
| Breeding | 10 | 296.820 | 298.61 | 4.89 | 0.00 | 17.09 | 1 | 99.96 | 88.24 | 74.65 | 29.20 | 2.82 | 0.39 |
| Breeding | 10 | 0.005 | 0.35 | 1.47 | 13.10 | 31.62 | 0 | 100.00 | 20.00 | 0.00 | 30.07 | 21.95 | 0.61 |
| Breeding | 10 | 0.469 | 16.22 | 6.68 | 0.11 | 22.99 | 40 | 98.06 | 78.90 | 34.15 | 29.60 | 13.81 | 1.45 |
| Breeding | 10 | 0.196 | 3.54 | 2.25 | 0.64 | 2.91 | 23 | 100.00 | 97.25 | 86.25 | 29.82 | 6.50 | 1.49 |
| Breeding | 10 | 8.200 | 76.23 | 7.51 | 0.64 | 2.44 | 19 | 95.98 | 96.71 | 88.90 | 30.36 | 4.90 | 2.09 |
| Breeding | 50 | 0.001 | 0.01 | 0.08 | 0.46 | 0.49 | 87 | 58.33 | 66.67 | 33.33 | 29.46 | 22.07 | 0.89 |
| Breeding | 50 | 0.095 | 2.60 | 2.39 | 0.06 | 27.76 | 474 | 89.52 | 82.86 | 39.05 | 29.76 | 4.07 | 2.74 |
| Breeding | 50 | 0.126 | 2.82 | 2.24 | 0.84 | 33.16 | 474 | 94.29 | 71.43 | 3.57 | 29.67 | 6.53 | 2.25 |
| Breeding | 50 | 8.040 | 118.54 | 11.80 | 0.09 | 5.50 | 419 | 99.38 | 92.54 | 76.37 | 30.23 | 16.75 | 2.41 |
| Breeding | 50 | 0.014 | 0.60 | 1.41 | 4.43 | 29.63 | 444 | 87.50 | 37.50 | 37.50 | 30.14 | 3.51 | 2.62 |
| Breeding | 50 | 0.047 | 1.72 | 2.24 | 0.08 | 29.20 | 493 | 96.15 | 78.85 | 40.38 | 30.20 | 2.67 | 1.47 |
| Breeding | 50 | 0.042 | 1.26 | 1.73 | 2.23 | 29.54 | 480 | 95.74 | 89.36 | 57.45 | 30.18 | 2.02 | 1.45 |
| Breeding | 50 | 0.019 | 0.83 | 1.70 | 0.39 | 22.23 | 471 | 90.48 | 85.71 | 61.90 | 29.76 | 11.28 | 1.65 |
| Breeding | 50 | 4.801 | 56.88 | 7.32 | 0.00 | 18.00 | 392 | 98.52 | 97.50 | 89.79 | 30.12 | 4.52 | 1.87 |
| Breeding | 50 | 0.059 | 1.72 | 2.01 | 0.24 | 6.09 | 360 | 92.31 | 78.46 | 49.23 | 29.58 | 7.37 | 1.32 |
| Breeding | 50 | 0.029 | 0.78 | 1.30 | 3.57 | 8.16 | 312 | 87.50 | 43.75 | 0.00 | 28.97 | 2.93 | 1.10 |
| Breeding | 50 | 0.023 | 1.24 | 2.29 | 2.37 | 33.46 | 398 | 76.92 | 92.31 | 3.85 | 30.14 | 4.03 | 1.08 |
| Breeding | 50 | 0.006 | 0.60 | 2.13 | 0.10 | 2.21 | 237 | 100.00 | 14.29 | 0.00 | 28.41 | 11.09 | 0.71 |
| Breeding | 50 | 0.038 | 0.86 | 1.24 | 0.15 | 5.97 | 271 | 79.69 | 66.67 | 9.38 | 29.51 | 8.11 | 0.94 |
| Breeding | 50 | 16.437 | 90.48 | 6.30 | 0.08 | 25.52 | 476 | 100.00 | 91.73 | 72.26 | 29.68 | 6.38 | 2.78 |
| Breeding | 50 | 0.026 | 0.84 | 1.47 | 6.01 | 30.53 | 429 | 100.00 | 86.21 | 51.72 | 28.85 | 2.28 | 1.40 |
| Breeding | 50 | 0.055 | 1.13 | 1.36 | 0.16 | 16.24 | 408 | 100.00 | 75.41 | 26.23 | 29.56 | 1.10 | 1.78 |
| Breeding | 50 | 0.083 | 1.56 | 1.53 | 0.13 | 4.67 | 182 | 94.57 | 97.83 | 86.96 | 29.54 | 7.58 | 2.43 |
| Breeding | 50 | 0.152 | 0.14 | 0.10 | 0.03 | 8.49 | 188 | 87.71 | 94.08 | 82.25 | 29.72 | 5.77 | 2.80 |
| Breeding | 50 | 0.134 | 0.91 | 0.70 | 0.10 | 6.24 | 182 | 75.84 | 26.85 | 6.71 | 29.54 | 5.65 | 2.40 |
| Breeding | 50 | 1.740 | 14.22 | 3.04 | 0.02 | 1.67 | 307 | 98.28 | 82.76 | 44.25 | 29.07 | 13.01 | 1.24 |
| Breeding | 50 | 14.350 | 44.15 | 3.29 | 0.03 | 0.00 | 251 | 91.44 | 81.95 | 49.76 | 29.43 | 6.60 | 1.19 |
| Breeding | 50 | 0.017 | 0.69 | 1.49 | 0.12 | 32.19 | 470 | 100.00 | 63.16 | 0.00 | 29.79 | 7.61 | 3.23 |
| Breeding | 50 | 1.632 | 8.43 | 1.86 | 0.08 | 12.88 | 573 | 98.63 | 97.08 | 91.23 | 29.60 | 3.24 | 1.92 |
| Breeding | 50 | 0.033 | 1.04 | 1.61 | 0.04 | 13.32 | 181 | 100.00 | 59.46 | 2.70 | 29.49 | 1.17 | 2.39 |
| Breeding | 50 | 7.362 | 27.81 | 2.89 | 0.00 | 6.74 | 191 | 98.75 | 97.83 | 91.44 | 29.44 | 5.42 | 2.46 |
| Breeding | 50 | 0.169 | 2.55 | 1.75 | 4.23 | 0.13 | 517 | 99.88 | 81.91 | 22.34 | 30.63 | 33.43 | 6.04 |
| Breeding | 50 | 0.002 | 0.24 | 1.60 | 3.91 | 37.83 | 784 | 100.00 | 50.00 | 0.00 | 30.16 | 19.26 | 1.07 |
| Breeding | 50 | 0.122 | 1.00 | 0.81 | 0.01 | 7.26 | 256 | 100.00 | 88.89 | 37.78 | 29.48 | 7.74 | 0.83 |
| Breeding | 50 | 60.550 | 36.03 | 1.31 | 0.40 | 1.30 | 145 | 98.65 | 97.08 | 86.97 | 30.62 | 81.13 | 1.67 |
| Breeding | 50 | 0.207 | 5.27 | 3.27 | 0.06 | 45.85 | 97 | 100.00 | 67.83 | 23.48 | 29.85 | 2.03 | 0.32 |
| Breeding | 50 | 23.370 | 193.17 | 11.27 | 0.00 | 58.49 | 328 | 96.53 | 92.38 | 68.16 | 29.95 | 3.01 | 0.48 |
| Breeding | 50 | 21.950 | 135.90 | 8.18 | 1.83 | 28.36 | 493 | 100.00 | 76.81 | 33.30 | 29.77 | 7.94 | 1.61 |
| Breeding | 50 | 2.224 | 25.80 | 4.88 | 0.02 | 24.65 | 82 | 96.24 | 91.46 | 40.64 | 29.95 | 3.42 | 1.94 |
| Breeding | 50 | 0.011 | 0.79 | 2.14 | 0.68 | 0.00 | 510 | 100.00 | 8.33 | 0.00 | 30.14 | 9.70 | 1.34 |
| Breeding | 50 | 9.073 | 47.72 | 4.47 | 0.01 | 19.16 | 89 | 99.64 | 93.50 | 72.22 | 29.91 | 2.98 | 1.87 |
| Breeding | 50 | 70.950 | 240.67 | 8.06 | 0.00 | 3.74 | 355 | 97.82 | 92.45 | 67.64 | 29.63 | 6.81 | 2.65 |
| Breeding | 50 | 0.155 | 0.61 | 0.44 | 0.04 | 0.47 | 156 | 87.79 | 90.70 | 73.26 | 29.75 | 1.89 | 1.41 |
| Breeding | 50 | 0.004 | 0.29 | 1.36 | 0.37 | 65.98 | 89 | 100.00 | 25.00 | 0.00 | 29.72 | 1.64 | 0.64 |
| Breeding | 50 | 0.014 | 0.95 | 2.31 | 1.13 | 28.18 | 483 | 100.00 | 40.00 | 0.00 | 29.85 | 4.07 | 3.78 |
| Breeding | 50 | 0.064 | 2.71 | 3.02 | 0.05 | 31.35 | 476 | 100.00 | 49.30 | 0.00 | 29.80 | 6.90 | 3.19 |
| Breeding | 50 | 0.005 | 0.56 | 2.36 | 0.17 | 30.93 | 483 | 100.00 | 0.00 | 0.00 | 29.81 | 6.57 | 3.14 |
| Breeding | 50 | 0.253 | 4.29 | 2.41 | 0.17 | 30.29 | 488 | 99.01 | 84.32 | 5.35 | 29.81 | 6.71 | 3.14 |
| Breeding | 50 | 296.820 | 298.61 | 4.89 | 0.00 | 17.09 | 429 | 99.96 | 88.24 | 74.65 | 29.82 | 2.82 | 2.44 |
| Breeding | 50 | 0.005 | 0.35 | 1.47 | 13.10 | 31.62 | 143 | 100.00 | 20.00 | 0.00 | 30.01 | 21.95 | 0.76 |
| Breeding | 50 | 0.469 | 16.22 | 6.68 | 0.11 | 22.99 | 482 | 98.06 | 78.90 | 34.15 | 29.00 | 13.81 | 2.06 |
| Breeding | 50 | 0.196 | 3.54 | 2.25 | 0.64 | 2.91 | 72 | 100.00 | 97.25 | 86.25 | 29.58 | 6.50 | 0.55 |
| Breeding | 50 | 8.200 | 76.23 | 7.51 | 0.64 | 2.44 | 67 | 95.98 | 96.71 | 88.90 | 30.16 | 4.90 | 0.60 |
| Breeding | 100 | 0.001 | 0.01 | 0.08 | 0.46 | 0.49 | 100 | 58.33 | 66.67 | 33.33 | 29.45 | 22.07 | 0.92 |
| Breeding | 100 | 0.095 | 2.60 | 2.39 | 0.06 | 27.76 | 890 | 89.52 | 82.86 | 39.05 | 29.53 | 4.07 | 1.62 |
| Breeding | 100 | 0.126 | 2.82 | 2.24 | 0.84 | 33.16 | 869 | 94.29 | 71.43 | 3.57 | 29.52 | 6.53 | 1.61 |
| Breeding | 100 | 8.040 | 118.54 | 11.80 | 0.09 | 5.50 | 872 | 99.38 | 92.54 | 76.37 | 30.02 | 16.75 | 1.20 |
| Breeding | 100 | 0.014 | 0.60 | 1.41 | 4.43 | 29.63 | 933 | 87.50 | 37.50 | 37.50 | 30.02 | 3.51 | 1.89 |
| Breeding | 100 | 0.047 | 1.72 | 2.24 | 0.08 | 29.20 | 919 | 96.15 | 78.85 | 40.38 | 30.02 | 2.67 | 1.17 |
| Breeding | 100 | 0.042 | 1.26 | 1.73 | 2.23 | 29.54 | 875 | 95.74 | 89.36 | 57.45 | 30.02 | 2.02 | 1.17 |
| Breeding | 100 | 0.019 | 0.83 | 1.70 | 0.39 | 22.23 | 810 | 90.48 | 85.71 | 61.90 | 29.70 | 11.28 | 1.26 |
| Breeding | 100 | 4.801 | 56.88 | 7.32 | 0.00 | 18.00 | 891 | 98.52 | 97.50 | 89.79 | 29.99 | 4.52 | 1.10 |
| Breeding | 100 | 0.059 | 1.72 | 2.01 | 0.24 | 6.09 | 304 | 92.31 | 78.46 | 49.23 | 29.51 | 7.37 | 1.08 |
| Breeding | 100 | 0.029 | 0.78 | 1.30 | 3.57 | 8.16 | 782 | 87.50 | 43.75 | 0.00 | 29.05 | 2.93 | 0.94 |
| Breeding | 100 | 0.023 | 1.24 | 2.29 | 2.37 | 33.46 | 1032 | 76.92 | 92.31 | 3.85 | 30.02 | 4.03 | 1.08 |
| Breeding | 100 | 0.006 | 0.60 | 2.13 | 0.10 | 2.21 | 215 | 100.00 | 14.29 | 0.00 | 28.39 | 11.09 | 0.59 |
| Breeding | 100 | 0.038 | 0.86 | 1.24 | 0.15 | 5.97 | 258 | 79.69 | 66.67 | 9.38 | 29.45 | 8.11 | 0.98 |
| Breeding | 100 | 16.437 | 90.48 | 6.30 | 0.08 | 25.52 | 896 | 100.00 | 91.73 | 72.26 | 29.36 | 6.38 | 1.59 |
| Breeding | 100 | 0.026 | 0.84 | 1.47 | 6.01 | 30.53 | 722 | 100.00 | 86.21 | 51.72 | 28.83 | 2.28 | 1.34 |
| Breeding | 100 | 0.055 | 1.13 | 1.36 | 0.16 | 16.24 | 1134 | 100.00 | 75.41 | 26.23 | 29.49 | 1.10 | 1.31 |
| Breeding | 100 | 0.083 | 1.56 | 1.53 | 0.13 | 4.67 | 1103 | 94.57 | 97.83 | 86.96 | 29.44 | 7.58 | 1.44 |
| Breeding | 100 | 0.152 | 0.14 | 0.10 | 0.03 | 8.49 | 1087 | 87.71 | 94.08 | 82.25 | 29.56 | 5.77 | 1.70 |
| Breeding | 100 | 0.134 | 0.91 | 0.70 | 0.10 | 6.24 | 1145 | 75.84 | 26.85 | 6.71 | 29.44 | 5.65 | 1.45 |
| Breeding | 100 | 1.740 | 14.22 | 3.04 | 0.02 | 1.67 | 274 | 98.28 | 82.76 | 44.25 | 29.03 | 13.01 | 1.00 |
| Breeding | 100 | 14.350 | 44.15 | 3.29 | 0.03 | 0.00 | 713 | 91.44 | 81.95 | 49.76 | 29.48 | 6.60 | 1.12 |
| Breeding | 100 | 0.017 | 0.69 | 1.49 | 0.12 | 32.19 | 899 | 100.00 | 63.16 | 0.00 | 29.61 | 7.61 | 2.01 |
| Breeding | 100 | 1.632 | 8.43 | 1.86 | 0.08 | 12.88 | 1231 | 98.63 | 97.08 | 91.23 | 29.48 | 3.24 | 1.28 |
| Breeding | 100 | 0.033 | 1.04 | 1.61 | 0.04 | 13.32 | 1067 | 100.00 | 59.46 | 2.70 | 29.39 | 1.17 | 1.55 |
| Breeding | 100 | 7.362 | 27.81 | 2.89 | 0.00 | 6.74 | 1068 | 98.75 | 97.83 | 91.44 | 29.28 | 5.42 | 1.52 |
| Breeding | 100 | 0.169 | 2.55 | 1.75 | 4.23 | 0.13 | 998 | 99.88 | 81.91 | 22.34 | 30.24 | 33.43 | 2.47 |
| Breeding | 100 | 0.002 | 0.24 | 1.60 | 3.91 | 37.83 | 1786 | 100.00 | 50.00 | 0.00 | 29.88 | 19.26 | 0.72 |
| Breeding | 100 | 0.122 | 1.00 | 0.81 | 0.01 | 7.26 | 252 | 100.00 | 88.89 | 37.78 | 29.43 | 7.74 | 0.96 |
| Breeding | 100 | 60.550 | 36.03 | 1.31 | 0.40 | 1.30 | 401 | 98.65 | 97.08 | 86.97 | 29.46 | 81.13 | 0.70 |
| Breeding | 100 | 0.207 | 5.27 | 3.27 | 0.06 | 45.85 | 611 | 100.00 | 67.83 | 23.48 | 29.78 | 2.03 | 0.59 |
| Breeding | 100 | 23.370 | 193.17 | 11.27 | 0.00 | 58.49 | 1471 | 96.53 | 92.38 | 68.16 | 29.78 | 3.01 | 0.60 |
| Breeding | 100 | 21.950 | 135.90 | 8.18 | 1.83 | 28.36 | 833 | 100.00 | 76.81 | 33.30 | 29.74 | 7.94 | 1.28 |
| Breeding | 100 | 2.224 | 25.80 | 4.88 | 0.02 | 24.65 | 353 | 96.24 | 91.46 | 40.64 | 29.71 | 3.42 | 1.45 |
| Breeding | 100 | 0.011 | 0.79 | 2.14 | 0.68 | 0.00 | 620 | 100.00 | 8.33 | 0.00 | 29.81 | 9.70 | 1.35 |
| Breeding | 100 | 9.073 | 47.72 | 4.47 | 0.01 | 19.16 | 348 | 99.64 | 93.50 | 72.22 | 29.71 | 2.98 | 1.35 |
| Breeding | 100 | 70.950 | 240.67 | 8.06 | 0.00 | 3.74 | 1233 | 97.82 | 92.45 | 67.64 | 29.46 | 6.81 | 1.56 |
| Breeding | 100 | 0.155 | 0.61 | 0.44 | 0.04 | 0.47 | 292 | 87.79 | 90.70 | 73.26 | 29.68 | 1.89 | 1.25 |
| Breeding | 100 | 0.004 | 0.29 | 1.36 | 0.37 | 65.98 | 648 | 100.00 | 25.00 | 0.00 | 29.59 | 1.64 | 0.78 |
| Breeding | 100 | 0.014 | 0.95 | 2.31 | 1.13 | 28.18 | 908 | 100.00 | 40.00 | 0.00 | 29.63 | 4.07 | 2.05 |
| Breeding | 100 | 0.064 | 2.71 | 3.02 | 0.05 | 31.35 | 977 | 100.00 | 49.30 | 0.00 | 29.61 | 6.90 | 2.03 |
| Breeding | 100 | 0.005 | 0.56 | 2.36 | 0.17 | 30.93 | 1040 | 100.00 | 0.00 | 0.00 | 29.61 | 6.57 | 2.04 |
| Breeding | 100 | 0.253 | 4.29 | 2.41 | 0.17 | 30.29 | 1056 | 99.01 | 84.32 | 5.35 | 29.61 | 6.71 | 2.04 |
| Breeding | 100 | 296.820 | 298.61 | 4.89 | 0.00 | 17.09 | 901 | 99.96 | 88.24 | 74.65 | 29.67 | 2.82 | 1.62 |
| Breeding | 100 | 0.005 | 0.35 | 1.47 | 13.10 | 31.62 | 904 | 100.00 | 20.00 | 0.00 | 29.83 | 21.95 | 0.63 |
| Breeding | 100 | 0.469 | 16.22 | 6.68 | 0.11 | 22.99 | 824 | 98.06 | 78.90 | 34.15 | 28.93 | 13.81 | 1.47 |
| Breeding | 100 | 0.196 | 3.54 | 2.25 | 0.64 | 2.91 | 161 | 100.00 | 97.25 | 86.25 | 29.76 | 6.50 | 0.99 |
| Breeding | 100 | 8.200 | 76.23 | 7.51 | 0.64 | 2.44 | 159 | 95.98 | 96.71 | 88.90 | 30.39 | 4.90 | 1.00 |
| Available | 10 | 14.350 | 44.15 | 3.29 | 0.03 | 0.00 | 8 | 98.82 | 88.78 | 55.26 | 30.09 | 6.60 | 2.90 |
| Available | 10 | 3.510 | 38.11 | 5.74 | 0.00 | 21.10 | 106 | 100.00 | 89.17 | 63.82 | 30.96 | 9.49 | 3.41 |
| Available | 10 | 0.130 | 2.37 | 1.86 | 1.67 | 1.21 | 31 | 92.52 | 79.86 | 19.44 | 31.43 | 31.30 | 8.25 |
| Available | 10 | 0.144 | 1.90 | 1.41 | 1.23 | 27.82 | 120 | 98.19 | 86.25 | 58.13 | 30.18 | 3.65 | 0.72 |
| Available | 10 | 0.004 | 0.39 | 1.83 | 0.08 | 29.47 | 78 | 100.00 | 75.00 | 0.00 | 30.14 | 2.57 | 0.76 |
| Available | 10 | 0.009 | 0.57 | 1.70 | 0.29 | 33.27 | 6 | 90.00 | 90.00 | 0.00 | 29.89 | 3.18 | 0.56 |
| Available | 10 | 0.016 | 0.71 | 1.57 | 0.02 | 32.00 | 5 | 100.00 | 88.89 | 66.67 | 29.91 | 4.04 | 0.53 |
| Available | 10 | 0.019 | 0.79 | 1.62 | 0.08 | 36.39 | 58 | 76.19 | 66.67 | 4.76 | 29.84 | 3.45 | 0.51 |
| Available | 10 | 0.005 | 0.34 | 1.43 | 0.73 | 37.92 | 57 | 80.00 | 60.00 | 0.00 | 29.82 | 2.99 | 0.48 |
| Available | 10 | 0.049 | 1.41 | 1.80 | 0.01 | 34.39 | 59 | 85.19 | 85.19 | 61.11 | 29.98 | 7.72 | 0.75 |
| Available | 10 | 1.481 | 13.35 | 3.10 | 0.03 | 32.50 | 49 | 100.00 | 86.49 | 43.28 | 29.92 | 6.36 | 0.69 |
| Available | 10 | 0.027 | 1.11 | 1.91 | 0.36 | 25.07 | 162 | 96.67 | 93.33 | 76.67 | 30.81 | 12.30 | 1.09 |
| Available | 10 | 0.631 | 4.81 | 1.71 | 0.35 | 26.98 | 47 | 100.00 | 96.72 | 90.16 | 30.29 | 10.89 | 1.30 |
| Available | 10 | 0.101 | 1.21 | 1.08 | 0.23 | 10.81 | 67 | 100.00 | 63.39 | 21.43 | 29.64 | 3.17 | 1.45 |
| Available | 10 | 278.789 | 197.01 | 3.33 | 0.01 | 0.00 | 21 | 99.95 | 96.29 | 85.65 | 29.35 | 0.95 | 0.62 |
| Available | 10 | 0.357 | 2.82 | 1.33 | 18.26 | 0.00 | 0 | 100.00 | 96.47 | 84.36 | 28.95 | 1.17 | 0.12 |
| Available | 10 | 0.104 | 1.68 | 1.47 | 0.20 | 1.73 | 20 | 92.24 | 87.93 | 62.93 | 32.58 | 70.08 | 6.88 |
| Available | 10 | 0.015 | 0.63 | 1.44 | 2.52 | 0.41 | 1 | 88.24 | 76.47 | 47.06 | 30.20 | 4.90 | 0.11 |
| Available | 10 | 0.032 | 1.00 | 1.57 | 0.84 | 30.86 | 848 | 94.44 | 94.44 | 83.33 | 31.15 | 7.24 | 4.66 |
| Available | 10 | 0.113 | 1.91 | 1.60 | 0.44 | 35.54 | 385 | 92.86 | 92.86 | 75.40 | 31.08 | 8.88 | 4.00 |
| Available | 10 | 0.012 | 0.61 | 1.59 | 0.02 | 46.58 | 129 | 84.62 | 84.62 | 61.54 | 30.81 | 2.23 | 2.05 |
| Available | 10 | 1.961 | 27.36 | 5.51 | 0.03 | 45.21 | 118 | 98.42 | 91.33 | 67.87 | 30.75 | 1.11 | 1.74 |
| Available | 10 | 2.102 | 26.27 | 5.11 | 0.42 | 46.65 | 76 | 99.01 | 92.86 | 70.98 | 30.69 | 2.38 | 0.86 |
| Available | 10 | 0.022 | 0.75 | 1.44 | 0.00 | 49.26 | 92 | 100.00 | 83.33 | 33.33 | 30.75 | 4.34 | 1.12 |
| Available | 10 | 0.598 | 8.49 | 3.10 | 7.98 | 37.96 | 100 | 99.85 | 95.34 | 87.81 | 30.96 | 9.06 | 2.29 |
| Available | 10 | 0.091 | 1.31 | 1.23 | 0.02 | 52.66 | 42 | 96.04 | 92.08 | 62.38 | 30.84 | 5.74 | 1.03 |
| Available | 10 | 0.015 | 0.85 | 1.94 | 0.57 | 46.45 | 8 | 100.00 | 52.94 | 5.88 | 30.98 | 18.54 | 0.74 |
| Available | 10 | 0.008 | 0.50 | 1.57 | 0.16 | 46.79 | 10 | 77.78 | 88.89 | 33.33 | 31.03 | 25.00 | 0.59 |
| Available | 10 | 0.013 | 0.60 | 1.51 | 0.20 | 22.77 | 31 | 85.71 | 85.71 | 57.14 | 31.49 | 62.39 | 1.07 |
| Available | 10 | 0.206 | 3.72 | 2.31 | 1.72 | 2.38 | 56 | 97.72 | 93.89 | 80.79 | 31.70 | 79.30 | 4.64 |
| Available | 10 | 0.014 | 0.68 | 1.60 | 1.19 | 6.32 | 16 | 100.00 | 93.75 | 50.00 | 31.54 | 69.90 | 3.56 |
| Available | 10 | 0.534 | 5.92 | 2.29 | 0.10 | 0.67 | 2 | 94.25 | 94.27 | 66.97 | 31.93 | 44.57 | 5.80 |
| Available | 10 | 5.380 | 31.34 | 3.81 | 0.64 | 5.72 | 86 | 100.00 | 92.75 | 62.27 | 31.53 | 32.15 | 4.47 |
| Available | 10 | 0.011 | 0.57 | 1.55 | 0.53 | 53.06 | 126 | 91.67 | 91.67 | 58.33 | 30.70 | 3.31 | 1.16 |
| Available | 10 | 0.033 | 1.22 | 1.89 | 0.20 | 51.24 | 70 | 91.89 | 81.08 | 56.76 | 30.55 | 5.58 | 1.07 |
| Available | 10 | 23.370 | 193.17 | 11.27 | 0.00 | 58.23 | 20 | 96.19 | 74.19 | 50.44 | 30.85 | 5.93 | 0.76 |
| Available | 10 | 5.610 | 21.93 | 2.61 | 0.06 | 0.00 | 12 | 100.00 | 92.51 | 73.44 | 30.63 | 3.37 | 1.06 |
| Available | 10 | 0.347 | 4.08 | 1.96 | 0.85 | 0.36 | 5 | 95.88 | 95.85 | 89.62 | 30.81 | 5.96 | 0.31 |
| Available | 10 | 0.067 | 1.38 | 1.51 | 2.29 | 0.68 | 6 | 98.65 | 95.95 | 86.49 | 31.10 | 31.39 | 7.99 |
| Available | 10 | 0.045 | 0.73 | 0.97 | 0.02 | 1.85 | 16 | 96.00 | 96.00 | 84.00 | 31.82 | 92.98 | 4.07 |
| Available | 10 | 0.002 | 0.12 | 0.80 | 0.11 | 21.26 | 83 | 5.56 | 0.00 | 0.00 | 30.95 | 11.14 | 2.79 |
| Available | 10 | 0.015 | 0.82 | 1.87 | 0.08 | 30.58 | 145 | 100.00 | 76.47 | 29.41 | 30.32 | 7.20 | 0.70 |
| Available | 10 | 0.005 | 0.37 | 1.42 | 0.23 | 24.10 | 52 | 83.33 | 66.67 | 33.33 | 30.73 | 15.03 | 1.36 |
| Available | 10 | 0.036 | 1.19 | 1.77 | 0.96 | 1.64 | 11 | 90.00 | 92.50 | 77.50 | 31.32 | 15.03 | 15.89 |
| Available | 10 | 23.090 | 65.37 | 3.84 | 0.16 | 24.87 | 66 | 99.61 | 95.84 | 82.11 | 30.32 | 10.00 | 1.40 |
| Available | 10 | 0.001 | 0.85 | 7.99 | 0.05 | 0.62 | 30 | 100.00 | 0.00 | 0.00 | 31.58 | 33.16 | 4.43 |
| Available | 10 | 0.002 | 0.21 | 1.40 | 0.59 | 26.39 | 170 | 100.00 | 0.00 | 0.00 | 30.72 | 10.91 | 0.85 |
| Available | 10 | 0.331 | 4.31 | 2.11 | 0.17 | 8.47 | 9 | 97.56 | 97.56 | 89.14 | 31.74 | 77.71 | 1.64 |
| Available | 10 | 0.022 | 1.01 | 1.94 | 0.09 | 40.31 | 3 | 100.00 | 91.67 | 75.00 | 31.01 | 30.80 | 0.58 |
| Available | 10 | 0.038 | 1.39 | 2.02 | 4.82 | 40.35 | 1 | 95.24 | 80.95 | 42.86 | 30.75 | 19.53 | 0.60 |
| Available | 10 | 0.556 | 4.31 | 1.63 | 0.97 | 3.88 | 10 | 97.37 | 97.57 | 88.82 | 29.90 | 4.01 | 1.85 |
| Available | 10 | 1.889 | 13.35 | 2.74 | 0.04 | 0.81 | 29 | 94.76 | 95.66 | 83.30 | 30.04 | 5.73 | 2.43 |
| Available | 50 | 14.350 | 44.15 | 3.29 | 0.03 | 0.00 | 251 | 98.82 | 88.78 | 55.26 | 29.82 | 6.60 | 1.19 |
| Available | 50 | 3.510 | 38.11 | 5.74 | 0.00 | 21.10 | 419 | 100.00 | 89.17 | 63.82 | 30.22 | 9.49 | 2.34 |
| Available | 50 | 0.130 | 2.37 | 1.86 | 1.67 | 1.21 | 582 | 92.52 | 79.86 | 19.44 | 30.48 | 31.30 | 2.34 |
| Available | 50 | 0.144 | 1.90 | 1.41 | 1.23 | 27.82 | 515 | 98.19 | 86.25 | 58.13 | 30.19 | 3.65 | 1.50 |
| Available | 50 | 0.004 | 0.39 | 1.83 | 0.08 | 29.47 | 514 | 100.00 | 75.00 | 0.00 | 30.19 | 2.57 | 1.46 |
| Available | 50 | 0.009 | 0.57 | 1.70 | 0.29 | 33.27 | 586 | 90.00 | 90.00 | 0.00 | 30.17 | 3.18 | 1.21 |
| Available | 50 | 0.016 | 0.71 | 1.57 | 0.02 | 32.00 | 560 | 100.00 | 88.89 | 66.67 | 30.18 | 4.04 | 1.27 |
| Available | 50 | 0.019 | 0.79 | 1.62 | 0.08 | 36.39 | 498 | 76.19 | 66.67 | 4.76 | 30.11 | 3.45 | 1.03 |
| Available | 50 | 0.005 | 0.34 | 1.43 | 0.73 | 37.92 | 482 | 80.00 | 60.00 | 0.00 | 30.09 | 2.99 | 1.00 |
| Available | 50 | 0.049 | 1.41 | 1.80 | 0.01 | 34.39 | 502 | 85.19 | 85.19 | 61.11 | 30.11 | 7.72 | 1.07 |
| Available | 50 | 1.481 | 13.35 | 3.10 | 0.03 | 32.50 | 522 | 100.00 | 86.49 | 43.28 | 30.08 | 6.36 | 1.06 |
| Available | 50 | 0.027 | 1.11 | 1.91 | 0.36 | 25.07 | 577 | 96.67 | 93.33 | 76.67 | 30.04 | 12.30 | 1.15 |
| Available | 50 | 0.631 | 4.81 | 1.71 | 0.35 | 26.98 | 449 | 100.00 | 96.72 | 90.16 | 29.95 | 10.89 | 1.07 |
| Available | 50 | 0.101 | 1.21 | 1.08 | 0.23 | 10.81 | 212 | 100.00 | 63.39 | 21.43 | 29.60 | 3.17 | 0.70 |
| Available | 50 | 278.789 | 197.01 | 3.33 | 0.01 | 0.00 | 194 | 99.95 | 96.29 | 85.65 | 29.42 | 0.95 | 0.56 |
| Available | 50 | 0.357 | 2.82 | 1.33 | 18.26 | 0.00 | 79 | 100.00 | 96.47 | 84.36 | 29.03 | 1.17 | 0.12 |
| Available | 50 | 0.104 | 1.68 | 1.47 | 0.20 | 1.73 | 144 | 92.24 | 87.93 | 62.93 | 31.66 | 70.08 | 1.73 |
| Available | 50 | 0.015 | 0.63 | 1.44 | 2.52 | 0.41 | 55 | 88.24 | 76.47 | 47.06 | 30.09 | 4.90 | 0.07 |
| Available | 50 | 0.032 | 1.00 | 1.57 | 0.84 | 30.86 | 1305 | 94.44 | 94.44 | 83.33 | 30.75 | 7.24 | 1.02 |
| Available | 50 | 0.113 | 1.91 | 1.60 | 0.44 | 35.54 | 1308 | 92.86 | 92.86 | 75.40 | 30.74 | 8.88 | 0.98 |
| Available | 50 | 0.012 | 0.61 | 1.59 | 0.02 | 46.58 | 1223 | 84.62 | 84.62 | 61.54 | 30.64 | 2.23 | 0.82 |
| Available | 50 | 1.961 | 27.36 | 5.51 | 0.03 | 45.21 | 1170 | 98.42 | 91.33 | 67.87 | 30.63 | 1.11 | 0.81 |
| Available | 50 | 2.102 | 26.27 | 5.11 | 0.42 | 46.65 | 760 | 99.01 | 92.86 | 70.98 | 30.65 | 2.38 | 0.70 |
| Available | 50 | 0.022 | 0.75 | 1.44 | 0.00 | 49.26 | 801 | 100.00 | 83.33 | 33.33 | 30.67 | 4.34 | 0.75 |
| Available | 50 | 0.598 | 8.49 | 3.10 | 7.98 | 37.96 | 1149 | 99.85 | 95.34 | 87.81 | 30.74 | 9.06 | 0.92 |
| Available | 50 | 0.091 | 1.31 | 1.23 | 0.02 | 52.66 | 674 | 96.04 | 92.08 | 62.38 | 30.70 | 5.74 | 0.72 |
| Available | 50 | 0.015 | 0.85 | 1.94 | 0.57 | 46.45 | 630 | 100.00 | 52.94 | 5.88 | 30.83 | 18.54 | 0.87 |
| Available | 50 | 0.008 | 0.50 | 1.57 | 0.16 | 46.79 | 559 | 77.78 | 88.89 | 33.33 | 30.92 | 25.00 | 0.98 |
| Available | 50 | 0.013 | 0.60 | 1.51 | 0.20 | 22.77 | 183 | 85.71 | 85.71 | 57.14 | 31.18 | 62.39 | 1.30 |
| Available | 50 | 0.206 | 3.72 | 2.31 | 1.72 | 2.38 | 152 | 97.72 | 93.89 | 80.79 | 31.26 | 79.30 | 1.47 |
| Available | 50 | 0.014 | 0.68 | 1.60 | 1.19 | 6.32 | 145 | 100.00 | 93.75 | 50.00 | 31.18 | 69.90 | 1.42 |
| Available | 50 | 0.534 | 5.92 | 2.29 | 0.10 | 0.67 | 395 | 94.25 | 94.27 | 66.97 | 30.84 | 44.57 | 3.51 |
| Available | 50 | 5.380 | 31.34 | 3.81 | 0.64 | 5.72 | 577 | 100.00 | 92.75 | 62.27 | 30.51 | 32.15 | 2.14 |
| Available | 50 | 0.011 | 0.57 | 1.55 | 0.53 | 53.06 | 194 | 91.67 | 91.67 | 58.33 | 30.57 | 3.31 | 0.48 |
| Available | 50 | 0.033 | 1.22 | 1.89 | 0.20 | 51.24 | 179 | 91.89 | 81.08 | 56.76 | 30.49 | 5.58 | 0.49 |
| Available | 50 | 23.370 | 193.17 | 11.27 | 0.00 | 58.23 | 336 | 96.19 | 74.19 | 50.44 | 30.66 | 5.93 | 0.53 |
| Available | 50 | 5.610 | 21.93 | 2.61 | 0.06 | 0.00 | 52 | 100.00 | 92.51 | 73.44 | 30.37 | 3.37 | 0.36 |
| Available | 50 | 0.347 | 4.08 | 1.96 | 0.85 | 0.36 | 13 | 95.88 | 95.85 | 89.62 | 30.44 | 5.96 | 0.11 |
| Available | 50 | 0.067 | 1.38 | 1.51 | 2.29 | 0.68 | 438 | 98.65 | 95.95 | 86.49 | 30.54 | 31.39 | 3.65 |
| Available | 50 | 0.045 | 0.73 | 0.97 | 0.02 | 1.85 | 132 | 96.00 | 96.00 | 84.00 | 31.58 | 92.98 | 1.49 |
| Available | 50 | 0.002 | 0.12 | 0.80 | 0.11 | 21.26 | 490 | 5.56 | 0.00 | 0.00 | 30.26 | 11.14 | 1.60 |
| Available | 50 | 0.015 | 0.82 | 1.87 | 0.08 | 30.58 | 558 | 100.00 | 76.47 | 29.41 | 30.03 | 7.20 | 1.02 |
| Available | 50 | 0.005 | 0.37 | 1.42 | 0.23 | 24.10 | 506 | 83.33 | 66.67 | 33.33 | 30.04 | 15.03 | 1.19 |
| Available | 50 | 0.036 | 1.19 | 1.77 | 0.96 | 1.64 | 194 | 90.00 | 92.50 | 77.50 | 29.51 | 15.03 | 0.83 |
| Available | 50 | 23.090 | 65.37 | 3.84 | 0.16 | 24.87 | 502 | 99.61 | 95.84 | 82.11 | 30.15 | 10.00 | 1.69 |
| Available | 50 | 0.001 | 0.85 | 7.99 | 0.05 | 0.62 | 458 | 100.00 | 0.00 | 0.00 | 30.56 | 33.16 | 2.34 |
| Available | 50 | 0.002 | 0.21 | 1.40 | 0.59 | 26.39 | 630 | 100.00 | 0.00 | 0.00 | 30.05 | 10.91 | 1.09 |
| Available | 50 | 0.331 | 4.31 | 2.11 | 0.17 | 8.47 | 151 | 97.56 | 97.56 | 89.14 | 31.42 | 77.71 | 1.42 |
| Available | 50 | 0.022 | 1.01 | 1.94 | 0.09 | 40.31 | 396 | 100.00 | 91.67 | 75.00 | 30.95 | 30.80 | 1.08 |
| Available | 50 | 0.038 | 1.39 | 2.02 | 4.82 | 40.35 | 260 | 95.24 | 80.95 | 42.86 | 30.80 | 19.53 | 0.91 |
| Available | 50 | 0.556 | 4.31 | 1.63 | 0.97 | 3.88 | 49 | 97.37 | 97.57 | 88.82 | 29.89 | 4.01 | 0.53 |
| Available | 50 | 1.889 | 13.35 | 2.74 | 0.04 | 0.81 | 52 | 94.76 | 95.66 | 83.30 | 29.89 | 5.73 | 0.57 |
| Available | 100 | 14.350 | 44.15 | 3.29 | 0.03 | 0.00 | 713 | 98.82 | 88.78 | 55.26 | 29.82 | 6.60 | 1.16 |
| Available | 100 | 3.510 | 38.11 | 5.74 | 0.00 | 21.10 | 878 | 100.00 | 89.17 | 63.82 | 29.99 | 9.49 | 1.27 |
| Available | 100 | 0.130 | 2.37 | 1.86 | 1.67 | 1.21 | 1036 | 92.52 | 79.86 | 19.44 | 30.14 | 31.30 | 1.46 |
| Available | 100 | 0.144 | 1.90 | 1.41 | 1.23 | 27.82 | 901 | 98.19 | 86.25 | 58.13 | 30.01 | 3.65 | 1.17 |
| Available | 100 | 0.004 | 0.39 | 1.83 | 0.08 | 29.47 | 922 | 100.00 | 75.00 | 0.00 | 30.02 | 2.57 | 1.17 |
| Available | 100 | 0.009 | 0.57 | 1.70 | 0.29 | 33.27 | 1026 | 90.00 | 90.00 | 0.00 | 30.02 | 3.18 | 1.07 |
| Available | 100 | 0.016 | 0.71 | 1.57 | 0.02 | 32.00 | 995 | 100.00 | 88.89 | 66.67 | 30.02 | 4.04 | 1.08 |
| Available | 100 | 0.019 | 0.79 | 1.62 | 0.08 | 36.39 | 995 | 76.19 | 66.67 | 4.76 | 30.01 | 3.45 | 1.07 |
| Available | 100 | 0.005 | 0.34 | 1.43 | 0.73 | 37.92 | 966 | 80.00 | 60.00 | 0.00 | 30.01 | 2.99 | 1.06 |
| Available | 100 | 0.049 | 1.41 | 1.80 | 0.01 | 34.39 | 957 | 85.19 | 85.19 | 61.11 | 30.02 | 7.72 | 1.09 |
| Available | 100 | 1.481 | 13.35 | 3.10 | 0.03 | 32.50 | 895 | 100.00 | 86.49 | 43.28 | 30.01 | 6.36 | 1.07 |
| Available | 100 | 0.027 | 1.11 | 1.91 | 0.36 | 25.07 | 854 | 96.67 | 93.33 | 76.67 | 29.98 | 12.30 | 1.00 |
| Available | 100 | 0.631 | 4.81 | 1.71 | 0.35 | 26.98 | 782 | 100.00 | 96.72 | 90.16 | 29.91 | 10.89 | 0.89 |
| Available | 100 | 0.101 | 1.21 | 1.08 | 0.23 | 10.81 | 476 | 100.00 | 63.39 | 21.43 | 29.62 | 3.17 | 0.71 |
| Available | 100 | 278.789 | 197.01 | 3.33 | 0.01 | 0.00 | 435 | 99.95 | 96.29 | 85.65 | 29.51 | 0.95 | 0.60 |
| Available | 100 | 0.357 | 2.82 | 1.33 | 18.26 | 0.00 | 182 | 100.00 | 96.47 | 84.36 | 29.23 | 1.17 | 0.30 |
| Available | 100 | 0.104 | 1.68 | 1.47 | 0.20 | 1.73 | 345 | 92.24 | 87.93 | 62.93 | 30.45 | 70.08 | 0.62 |
| Available | 100 | 0.015 | 0.63 | 1.44 | 2.52 | 0.41 | 128 | 88.24 | 76.47 | 47.06 | 29.77 | 4.90 | 0.16 |
| Available | 100 | 0.032 | 1.00 | 1.57 | 0.84 | 30.86 | 1702 | 94.44 | 94.44 | 83.33 | 30.60 | 7.24 | 0.64 |
| Available | 100 | 0.113 | 1.91 | 1.60 | 0.44 | 35.54 | 1754 | 92.86 | 92.86 | 75.40 | 30.59 | 8.88 | 0.68 |
| Available | 100 | 0.012 | 0.61 | 1.59 | 0.02 | 46.58 | 1768 | 84.62 | 84.62 | 61.54 | 30.51 | 2.23 | 0.58 |
| Available | 100 | 1.961 | 27.36 | 5.51 | 0.03 | 45.21 | 1768 | 98.42 | 91.33 | 67.87 | 30.50 | 1.11 | 0.58 |
| Available | 100 | 2.102 | 26.27 | 5.11 | 0.42 | 46.65 | 1707 | 99.01 | 92.86 | 70.98 | 30.49 | 2.38 | 0.58 |
| Available | 100 | 0.022 | 0.75 | 1.44 | 0.00 | 49.26 | 1744 | 100.00 | 83.33 | 33.33 | 30.50 | 4.34 | 0.60 |
| Available | 100 | 0.598 | 8.49 | 3.10 | 7.98 | 37.96 | 1804 | 99.85 | 95.34 | 87.81 | 30.55 | 9.06 | 0.66 |
| Available | 100 | 0.091 | 1.31 | 1.23 | 0.02 | 52.66 | 1673 | 96.04 | 92.08 | 62.38 | 30.50 | 5.74 | 0.60 |
| Available | 100 | 0.015 | 0.85 | 1.94 | 0.57 | 46.45 | 1691 | 100.00 | 52.94 | 5.88 | 30.55 | 18.54 | 0.66 |
| Available | 100 | 0.008 | 0.50 | 1.57 | 0.16 | 46.79 | 1704 | 77.78 | 88.89 | 33.33 | 30.57 | 25.00 | 0.70 |
| Available | 100 | 0.013 | 0.60 | 1.51 | 0.20 | 22.77 | 1700 | 85.71 | 85.71 | 57.14 | 30.80 | 62.39 | 1.14 |
| Available | 100 | 0.206 | 3.72 | 2.31 | 1.72 | 2.38 | 1515 | 97.72 | 93.89 | 80.79 | 30.90 | 79.30 | 1.57 |
| Available | 100 | 0.014 | 0.68 | 1.60 | 1.19 | 6.32 | 1220 | 100.00 | 93.75 | 50.00 | 30.83 | 69.90 | 1.39 |
| Available | 100 | 0.534 | 5.92 | 2.29 | 0.10 | 0.67 | 1124 | 94.25 | 94.27 | 66.97 | 30.28 | 44.57 | 1.53 |
| Available | 100 | 5.380 | 31.34 | 3.81 | 0.64 | 5.72 | 1146 | 100.00 | 92.75 | 62.27 | 30.16 | 32.15 | 1.25 |
| Available | 100 | 0.011 | 0.57 | 1.55 | 0.53 | 53.06 | 1141 | 91.67 | 91.67 | 58.33 | 30.43 | 3.31 | 0.53 |
| Available | 100 | 0.033 | 1.22 | 1.89 | 0.20 | 51.24 | 914 | 91.89 | 81.08 | 56.76 | 30.41 | 5.58 | 0.47 |
| Available | 100 | 23.370 | 193.17 | 11.27 | 0.00 | 58.23 | 1524 | 96.19 | 74.19 | 50.44 | 30.46 | 5.93 | 0.57 |
| Available | 100 | 5.610 | 21.93 | 2.61 | 0.06 | 0.00 | 151 | 100.00 | 92.51 | 73.44 | 30.39 | 3.37 | 0.62 |
| Available | 100 | 0.347 | 4.08 | 1.96 | 0.85 | 0.36 | 182 | 95.88 | 95.85 | 89.62 | 30.38 | 5.96 | 0.75 |
| Available | 100 | 0.067 | 1.38 | 1.51 | 2.29 | 0.68 | 936 | 98.65 | 95.95 | 86.49 | 30.15 | 31.39 | 1.54 |
| Available | 100 | 0.045 | 0.73 | 0.97 | 0.02 | 1.85 | 454 | 96.00 | 96.00 | 84.00 | 30.58 | 92.98 | 0.88 |
| Available | 100 | 0.002 | 0.12 | 0.80 | 0.11 | 21.26 | 1017 | 5.56 | 0.00 | 0.00 | 30.03 | 11.14 | 1.19 |
| Available | 100 | 0.015 | 0.82 | 1.87 | 0.08 | 30.58 | 841 | 100.00 | 76.47 | 29.41 | 29.99 | 7.20 | 1.02 |
| Available | 100 | 0.005 | 0.37 | 1.42 | 0.23 | 24.10 | 803 | 83.33 | 66.67 | 33.33 | 29.98 | 15.03 | 0.99 |
| Available | 100 | 0.036 | 1.19 | 1.77 | 0.96 | 1.64 | 507 | 90.00 | 92.50 | 77.50 | 29.60 | 15.03 | 0.71 |
| Available | 100 | 23.090 | 65.37 | 3.84 | 0.16 | 24.87 | 895 | 99.61 | 95.84 | 82.11 | 29.99 | 10.00 | 1.21 |
| Available | 100 | 0.001 | 0.85 | 7.99 | 0.05 | 0.62 | 1095 | 100.00 | 0.00 | 0.00 | 30.19 | 33.16 | 1.39 |
| Available | 100 | 0.002 | 0.21 | 1.40 | 0.59 | 26.39 | 854 | 100.00 | 0.00 | 0.00 | 29.99 | 10.91 | 1.02 |
| Available | 100 | 0.331 | 4.31 | 2.11 | 0.17 | 8.47 | 626 | 97.56 | 97.56 | 89.14 | 30.61 | 77.71 | 0.82 |
| Available | 100 | 0.022 | 1.01 | 1.94 | 0.09 | 40.31 | 1669 | 100.00 | 91.67 | 75.00 | 30.57 | 30.80 | 0.73 |
| Available | 100 | 0.038 | 1.39 | 2.02 | 4.82 | 40.35 | 1283 | 95.24 | 80.95 | 42.86 | 30.51 | 19.53 | 0.64 |
| Available | 100 | 0.556 | 4.31 | 1.63 | 0.97 | 3.88 | 157 | 97.37 | 97.57 | 88.82 | 30.15 | 4.01 | 1.06 |
| Available | 100 | 1.889 | 13.35 | 2.74 | 0.04 | 0.81 | 164 | 94.76 | 95.66 | 83.30 | 30.15 | 5.73 | 1.06 |
